# Dual transcriptional analysis reveals adaptation of host and pathogen to intracellular survival of *Pseudomonas aeruginosa* associated with urinary tract infection

**DOI:** 10.1371/journal.ppat.1009534

**Published:** 2021-04-26

**Authors:** Cristina Penaranda, Nicole M. Chumbler, Deborah T. Hung

**Affiliations:** 1 Infectious Disease and Microbiome Program, Broad Institute of Harvard and MIT, Cambridge, Massachusetts, United States of America; 2 Department of Molecular Biology and Center for Computational and Integrative Biology, Massachusetts General Hospital, Boston, Massachusetts, United States of America; 3 Department of Genetics, Harvard Medical School, Boston, Massachusetts, United States of America; Universite de Reims Champagne-Ardenne, FRANCE

## Abstract

Long-term survival of bacterial pathogens during persistent bacterial infections can be associated with antibiotic treatment failure and poses a serious public health problem. Infections caused by the Gram-negative pathogen *Pseudomonas aeruginosa*, which can cause both acute and chronic infections, are particularly challenging due to its high intrinsic resistance to antibiotics. The ineffectiveness of antibiotics is exacerbated when bacteria reside intracellularly within host cells where they can adopt a drug tolerant state. While the early steps of adherence and entry of *P*. *aeruginosa* into mammalian cells have been described, the subsequent fate of internalized bacteria, as well as host and bacterial molecular pathways facilitating bacterial long-term survival, are not well defined. In particular, long-term survival within bladder epithelial cells has not been demonstrated and this may have important implications for the understanding and treatment of UTIs caused by *P*. *aeruginosa*. Here, we demonstrate and characterize the intracellular survival of wild type (WT) *P*. *aeruginosa* inside bladder epithelial cells and a mutant with a disruption in the bacterial two-component regulator AlgR that is unable to survive intracellularly. Using simultaneous dual RNA-seq transcriptional profiling, we define the transcriptional response of intracellular bacteria and their corresponding invaded host cells. The bacterial transcriptional response demonstrates that WT bacteria rapidly adapt to the stress encountered in the intracellular environment in contrast to *ΔalgR* bacteria. Analysis of the host transcriptional response to invasion suggests that the NF-κB signaling pathway, previously shown to be required for extracellular bacterial clearance, is paradoxically also required for intracellular bacterial survival. Lastly, we demonstrate that intracellular survival is important for pathogenesis of *P*. *aeruginosa in vivo* using a model of murine urinary tract infection. We propose that the unappreciated ability of *P*. *aeruginosa* to survive intracellularly may play an important role in contributing to the chronicity and recurrence of *P*. *aeruginosa* in urinary tract infections.

## Author summary

Chronic persistent bacterial infections are a serious and growing public health problem worsened by the rise in antibiotic resistance, yet new approaches for treating these infections are lacking. These long-term infections can occur when bacteria invade and survive inside host cells where they can hide from the immune system and become less susceptible to killing by antibiotics. *Pseudomonas aeruginosa*, a bacterium conventionally considered an extracellular pathogen, can cause chronic infections of many organ systems, including the urinary tract. Here, we show that *P*. *aeruginosa* can in fact survive inside bladder epithelial cells and becomes tolerant to antibiotic treatment. Using gene expression analysis, we show that bacteria quickly adapt to the intracellular environment while the corresponding host cells upregulate the NF-κB signaling pathway. We demonstrate that this response, which had previously been shown to be required for clearance of extracellular bacteria, is paradoxically also required for survival of intracellular bacteria. We propose that the ability of *P*. *aeruginosa* to survive intracellularly plays an important role in contributing to the chronicity and recurrence of *P*. *aeruginosa* infections and that targeting host pathways, such as NF-κB signaling, could transform our ability to manage chronic and/or recurrent infections.

## Introduction

Persistent bacterial infections result from ineffective clearance by the host, often in conjunction with incomplete eradication by antimicrobial therapy. Persistent bacteria can result in chronic, indolent infection or repeated recrudescence of acute infection. The antibiotic tolerant state of these bacteria contributes to treatment failure, thus complicating management of these infections [1]. Greater insight into the host and pathogen molecular pathways enabling the establishment of persistent bacterial reservoirs is needed to devise new strategies to better manage these types of infections.

Urinary tract infections (UTIs) are one of the most prevalent bacterial infections resulting in major medical expenses each year with high rates of recurrence [2]. Among uropathogens, uropathogenic *E*. *coli* (UPEC) has been shown, both *in vivo* and *in vitro*, to invade bladder epithelial cells and form biofilm-like pods and quiescent intracellular reservoirs (QIRs) [3–5]. Intracellular bacteria are able to evade immune clearance, avoid killing by antibiotics, and form reservoirs that enable the recurrence of infections and the development of antibiotic resistance [6]. *Pseudomonas aeruginosa*, another important Gram-negative uropathogen, is the third most common cause of UTIs. Although most infections are nosocomial or catheter-associated [7], it is also prevalent in community-acquired infections, particularly in children [8]. Moreover, infection by *P*. *aeruginosa* is often difficult to treat and eradicate because of its intrinsic antibiotic resistance and increasing rates of resistance to existing antibiotics [9]. Although it has been demonstrated to invade epithelial and mast cells [10–12], little is known about the fate of internalized *P*. *aeruginosa* and whether, like UPEC, formation of stable intracellular reservoirs in the urinary epithelium has similar implications for persistent UTIs [13].

The interaction between *P*. *aeruginosa* and the mammalian host has been characterized both *in vivo* and *in vitro*. The bacterial transcriptional response is predominated by induction of the type III secretion system, which injects effector proteins into host cells [14], and upregulation of siderophores involved in iron sequestration [15]. Efficient clearance of extracellular bacteria relies on both epithelial and innate immune cells sensing *P*. *aeruginosa* through Toll-like receptor (TLR) recognition of pathogen-associated molecular patterns and downstream signaling through these receptors [16]. In murine models of pneumonia, inhibition of NF-κB signaling in epithelial cells resulted in impaired bacterial clearance leading to the conclusion that activation of NF-κB signaling is protective during *P*. *aeruginosa* infection [17]. However, this insight into the bacterial and host signaling pathways modulated during infection comes from bulk studies that aggregate analysis of intracellular and extracellular bacteria, as well as host cells that are invaded and not invaded but exposed to bacteria, resulting in a signal that is dominated by the response to extracellular bacteria. Identifying bacterial signaling pathways important for adaptation to the intracellular environment and host signaling pathways activated by intracellular bacteria may provide us with novel strategies to overcome chronic, persistent *P*. *aeruginosa* infections.

In the present study, we demonstrate that *P*. *aeruginosa* can survive intracellularly in bladder epithelial cells to form a stable intracellular reservoir and identify a bacterial mutant, *ΔalgR*, that is unable to survive intracellularly. Using dual RNA-seq transcriptional profiling, we define the bacterial and host transcriptional adaptations that occur during the early stage of this host-pathogen interaction leading to bacterial survival versus clearance. Our results indicate that intracellular bacterial survival is dependent on bacterial intrinsic mechanisms of adaptation to the intracellular environment rather than the specific induction of host clearance pathways. Importantly, induction of NF-κB signaling in invaded host cells is required for bacterial survival rather than clearance, which contrasts with the role of NF-κB signaling in clearance of extracellular bacteria. Intracellular survival in our *in vitro* model correlates with bladder colonization in a mouse model of UTI, suggesting that intracellular survival is critical for *in vivo* pathogenesis of *P*. *aeruginosa*. Finally, we demonstrate that these intracellular bacteria are antibiotic tolerant. Given its high intrinsic antibiotic resistance, the ability to form an intracellular reservoir during infection may contribute to poor eradication of *P*. *aeruginosa* even after antibiotic treatment, resulting in development of chronic or recurrent UTI infections.

## Results

### Pseudomonas aeruginosa forms a stable intracellular reservoir within bladder epithelial cells

**Fig 1:**
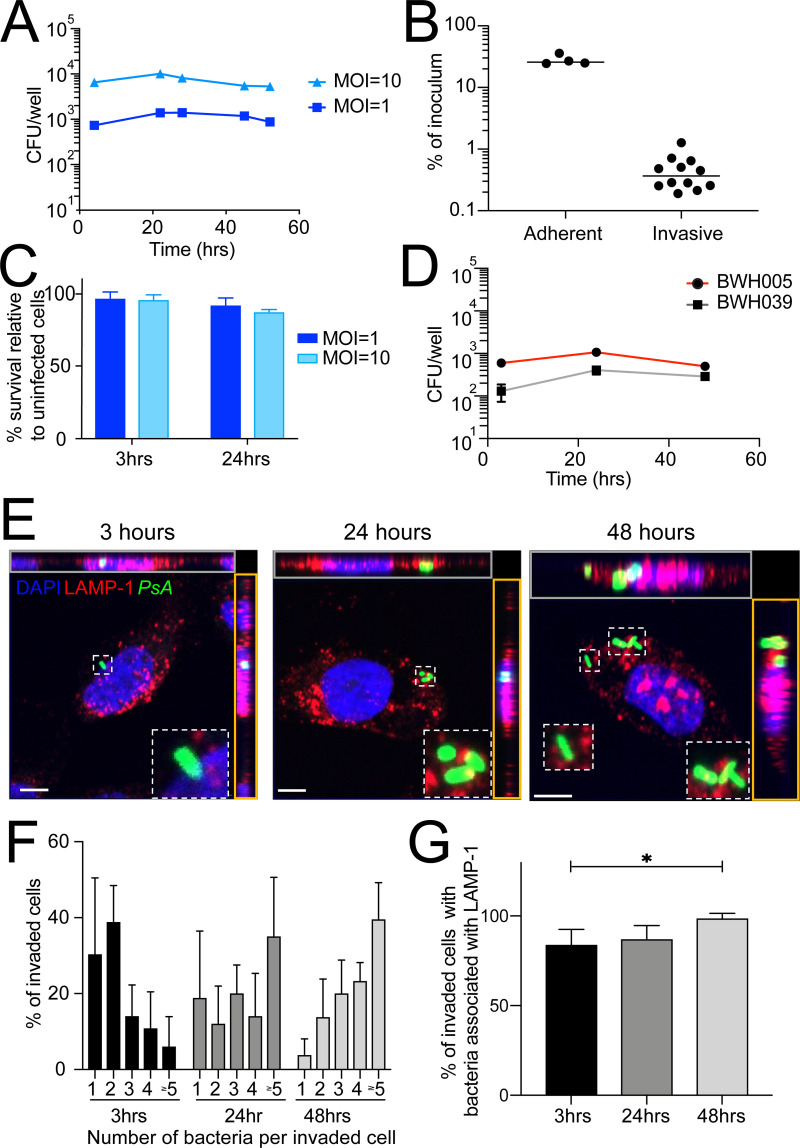
*Pseudomonas aeruginosa* survives intracellularly. A gentamicin protection assay was used to quantify intracellular bacterial burden. (A) Cells were infected with WT PAO1 at MOI = 1 or 10. (B) Host cell survival at 3 and 24 hpi with WT bacteria at MOI = 1 or 10 relative to uninfected cells harvested at the same time point. (C) Cells were infected at MOI = 1 with two clinical strains (BWH005 and BWH039) which were isolated from urine. (D) Cells were infected at MOI = 10 for 2 hours and incubated in the absence, to measure adherent bacteria, or presence of gentamicin, to measure invading bacteria, and harvested at 3 hpi. (E-G) Cells were infected with WT GFP-expressing bacteria at MOI = 10, fixed at the indicated time points and stained with anti-LAMP-1 antibodies and DAPI. (E) Representative images of invaded cells. Maximum intensity projections of XY (middle), and orthogonal XZ (gray outline) and YZ (orange outline) projections of the serial confocal images are shown. Scale bar = 5μm. (F) Intracellular bacterial load as determined by microscopy. (G) Percent of invaded cells with LAMP-1-associated bacteria. Data is representative of 4–6 biological replicates with at least 50 cells per condition. Error bars represent standard deviation. Data were analyzed by one-way ANOVA (p = 0.02).

Most studies of *P*. *aeruginosa* interaction with host cells have focused on mechanisms of bacterial adherence to, invasion of, and short term (<12 hours) survival within host cells [10–12,18], rather than the long-term fate of internalized bacteria. Moreover, long-term survival within bladder epithelial cells has not been demonstrated and this may have important implications for the understanding and treatment of UTIs caused by *P*. *aeruginosa*. To demonstrate that *P*. *aeruginosa* is able to establish stable intracellular reservoirs within host cells, we infected the human bladder epithelial cell line 5637 with the wild-type (WT) laboratory strain, PAO1, at a multiplicity of infection (MOI) of 1–10 and used a gentamicin protection assay followed by plating for colony forming units (CFU) to quantify intracellular bacterial load at various time points post-infection while preventing subsequent cycles of infection from free bacteria. This assay has been widely used to assess the invasion and intracellular survival of *P*. *aeruginosa* in various cell types [10,12,18]. We confirmed that the concentrations of gentamicin used prevents growth of *P*. *aeruginosa* in cell culture media (S1 Fig). We found that the intracellular bacterial load remained stable over 52 hours, as has been shown previously [12], demonstrating that PAO1 is able to survive within host cells (Fig 1A). While roughly 25% of bacteria were able to adhere to the epithelial cells, only ~0.5% were able to invade and survive intracellularly at 3 hours post infection (hpi), suggesting that invasion is a rare event (Fig 1B). In contrast to the cytotoxicity observed for PAO1 in other host cell types [19], PAO1 did not result in decreased survival of 5637 cells at 3 and 24 hpi compared to uninfected control cells (Fig 1C). This lack of host cell death may be attributed to a combination of the particular isolate of PAO1 [20], the host cell type [21] and the low MOI (1–10) used. Notably, intracellular survival was not restricted to this laboratory-adapted strain, as clinical strains isolated from the urine of patients (which also did not induce host cell death and thus were amenable to characterization) were also able to establish stable intracellular reservoirs lasting 48 hours (Figs 1D, S2A and S2B). Importantly, PAO1 was also able to survive in A459 lung epithelial cells, showing that intracellular survival can occur in multiple epithelial cell types which originate from the mucosal surfaces of different organs (S2C Fig). In contrast, intracellular bacterial load decreased more than 100-fold over 48 hours in the macrophage-like cell line J774 (S2D Fig).

To understand the replication and survival dynamics of intracellular *P*. *aeruginosa*, we used microscopy to determine intracellular bacterial load on a per cell basis over time. Importantly, in this analysis, bacteria colocalized with LAMP-1, an intracellular marker of endosomes/lysosomes, suggesting that they were indeed intracellular and not on the surface of the cell. At 3 hpi, the great majority of invaded cells (69%) harbored 1–2 intracellular bacteria, 25% of cells harbored 3–4 bacteria and only 6% of cells harbored ≥5 bacteria (Fig 1E and 1F). By 24 hpi, the fraction of cells harboring 1–2 bacteria decreased to 31%, while 34% of cells now harbored 3–4 bacteria and nearly 35% of cells harbored ≥5 bacteria (Fig 1F). Finally, by 48 hpi, only 17% of cells harbored 1–2 bacteria, while 43% of cells harbored 3–4 bacteria and 40% of cells harbored ≥5 bacteria (Fig 1F); of note, we did not find any cells harboring ≥10 bacteria. The shift in the percentage of cells containing 1–2 to ≥5 bacteria per cell overtime suggests intracellular bacterial proliferation. The slow proliferation rate, however, of ~3 doublings over 48 hours (doubling time of ~16 hours) is in stark contrast to the fast proliferation rate of planktonic *P*. *aeruginosa* in culture (doubling time of ~30 minutes [22]) or other intracellular pathogens, such as *Salmonella* typhimurium that can reach over 100 bacteria per epithelial cell within 10 hpi (doubling time of ~85 minutes [23]). The percentage of bacteria that colocalized with the endosomal/lysosomal marker LAMP-1 increased throughout infection, from 84% at 3 hpi to 99% at 48 hpi (Fig 1G). This finding suggests that residence within the endosome/lysosome may be required for intracellular survival as has been demonstrated for other intracellular pathogens [24].

Measuring the fraction of total cells harboring intracellular bacteria by microscopy, even at early time points, was not possible because of the rarity of this event. Therefore, we instead made a theoretical calculation to estimate the percentage of invaded cells. Using the number of host cells in the well at the time of infection, the total bacterial load per well determined by CFU (Fig 1A), and an average number of bacteria per cell determined by microscopy (2 at 3hpi, Fig 1F), we estimate that 0.5% of cells harbor bacteria at 3 hpi, which is consistent with the low level of UPEC invasion of the same cell line [25]. This cell line continues to proliferate during the assay, with the total number of cells per well increasing ~2.5-fold over the course of 48 hours. Taking into account that the total bacterial load per well remained constant (Fig 1A) while the number of host cells and the average number of bacteria per cell (4 at 48hpi, Fig 1F) both increased, we estimate that 0.1% of cells harbor bacteria at 48 hpi. This estimation assumes all bacteria that successfully invade are able to proliferate. However, our results demonstrate that heterogeneity in infection outcome exists such that some host cells are able to control proliferation (harboring 1 bacterium at 48 hpi), others support a low level of intracellular bacterial proliferation (harboring 2+ bacteria at 48 hpi), and others are likely able to clear bacteria (not measured in our analysis) resulting in a net bacterial load per well that remains constant overtime. Temporal studies that follow the fate of intracellular bacteria on a per cell basis are needed to more fully understand this heterogeneity.

### The bacterial transcriptional regulator AlgR is required for intracellular survival

**Fig 2:**
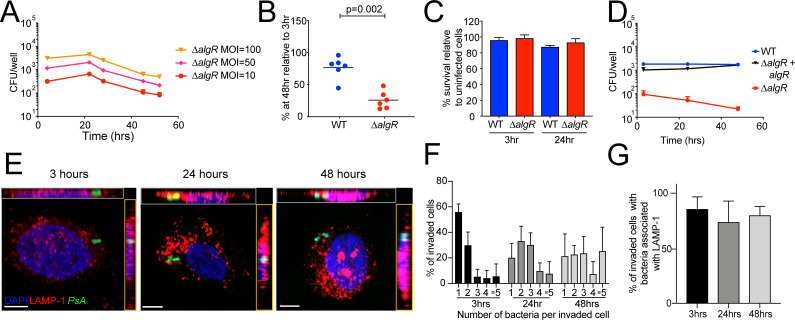
The bacterial two-component response regulator AlgR is required for intracellular survival of *Pseudomonas aeruginosa*. (A) Cells were infected with increasing MOIs of *ΔalgR* bacteria and intracellular bacterial load was determined. (B) Percent of recovered bacteria at 48 hpi relative to 3 hpi with MOI = 10. Each data point represents an independent experiment. Data were analyzed by unpaired T-test. (C) Host cell survival was measured at 3 and 24 hpi with WT or *ΔalgR* bacteria at MOI = 10 relative to uninfected cells harvested at the same time point. (D) *algR* was complemented in the *ΔalgR* deletion strain and cells were infected and harvested as in A. (E-F) Cells were infected with *ΔalgR* GFP-expressing bacteria at MOI = 10, fixed at the indicated time points and stained with anti-LAMP-1 antibodies and DAPI. (E) Representative images of invaded cells. Maximum intensity projections of XY (middle), and orthogonal XZ (gray outline) and YZ (orange outline) projections of the serial confocal images are shown. Scale bar = 5μm. (F) Intracellular bacterial load as determined by microscopy. (G) Percent of invaded cells with LAMP-1-associated bacteria. Data is representative of 4–6 biological replicates with at least 50 cells per condition. Error bars represent standard deviation.

Taking advantage of this model, we tested various mutants containing disruptions of regulatory factors to identify bacterial pathways required for intracellular survival of *P*. *aeruginosa*. We identified a mutant harboring an in-frame nonpolar deletion of *algR* that resulted in decreased intracellular bacterial survival, as determined by the lower fraction of mutant bacteria recovered at 48 hpi relative to 3 hpi, compared to WT bacteria (Figs 1A, 2A and 2B). AlgR is a member of the LytTR family of two-component response regulators that controls the expression of more than 200 genes [26]. It is a master regulator of virulence in both acute and chronic models of infection, and has been shown to positively regulate swimming motility, alginate production, and type IV pilin assembly and export, while negatively regulating quorum sensing, biofilm formation, type III secretion and anaerobic metabolism [27–35]. Because the type IV pilus is required for maximal adherence to and invasion of host cells [36], we examined the impact of decreased invasion of *ΔalgR* on the ability of bacteria to survive intracellularly. As expected, at the same MOI, infection with *ΔalgR* bacteria resulted in fewer invading bacteria at 3 hpi compared to WT bacteria (Figs 1A and 2A). To test whether intracellular survival was the result of an invasion bottleneck, we increased the number of invading mutant bacteria by increasing the MOI to obtain an equivalent or higher number of initial intracellular bacteria as WT at MOI 1; we still observed a pronounced defect in intracellular survival (Fig 2A). This decrease in recovered CFU for *ΔalgR* bacteria was not due to increased host cell death as a means to eliminate invaded host cells (Fig 2C). Importantly, the decrease in intracellular survival was also not due to generalized lower fitness of this mutant as it grew identically to WT in LB and M9 minimal media and was not more susceptible to antibiotics or sodium hypochlorite in culture, with the latter chosen to mimic the antimicrobial molecule produced intracellularly by host cells (S3 Fig). Both intracellular survival and invasion were restored to WT levels upon episomal complementation with *algR* in the *ΔalgR* background (Fig 2D). Thus, AlgR plays a role in both invasion and intracellular survival.

We used microscopy to characterize the replication and survival dynamics of *ΔalgR* bacteria (Fig 2E). At 3 hpi, the majority of *ΔalgR* invaded cells (56%) harbored 1 intracellular bacterium (Fig 2F). Based on imaging, the main difference between WT and *ΔalgR* bacteria is that intracellular *ΔalgR* bacteria proliferate to a lesser extent than WT bacteria with only 25% of cells harboring ≥5 bacteria at 48 hpi compared to 40% for WT bacteria (Figs 1F and 2F); this could be due to a slower doubling time or a longer lag time. In contrast to our findings with WT bacteria, the proportion of *ΔalgR* bacteria that colocalized with LAMP-1 did not increase throughout the time course of infection (Fig 2G). Nevertheless, the majority (>80%) of bacteria also remained confined to endosomes/lysosomes (Fig 2G) demonstrating that *ΔalgR* bacteria are able to reside within these intracellular compartments.

**Fig 3:**
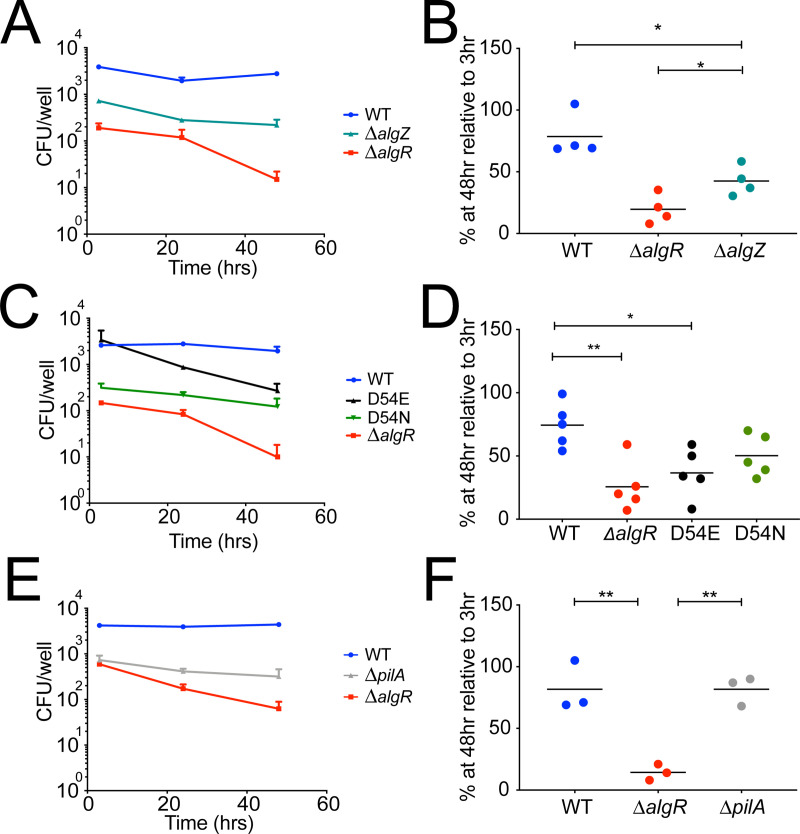
Phosphorylation-independent and -dependent roles of AlgR in intracellular survival and host cell invasion. Cells were infected at MOI = 10 with the kinase deficient strain *ΔalgZ*, the phosphomimetic D54E or non-phosphorylatable D54N single point *algR* mutants, or the pilus deficient strain *ΔpilA*. (A, C, E) At indicated time points, cells were lysed and the intracellular bacterial load was determined. (B, D, F) Percent of recovered bacteria at 48 hpi relative to 3 hpi. Each data point represents an independent experiment. *p<0.05 **p<0.005 based on one-way ANOVA.

Consistent with two-component regulator systems, phosphorylation of AlgR, likely by the histidine kinase AlgZ (FimS) can alter its function [37]. Phosphorylated AlgR positively regulates twitching motility through upregulation of the *fimU* prepilin cluster but is not required for the activation of alginate production [33,38]. To determine the role of AlgR phosphorylation in invasion and intracellular survival, we constructed an in-frame nonpolar deletion of *algZ* that would result in the absence of AlgR phosphorylation. We found that the *ΔalgZ* strain showed both decreased invasion and intracellular survival, although it did not phenocopy the *ΔalgR* strain (Fig 3A and 3B). Transcriptional analysis revealed that *algR* expression was decreased in the *ΔalgZ* strain (S3A Fig), raising the possibility that the overall decrease in total AlgR, rather than the specific absence of phosphorylated AlgR, is responsible for this discrepancy.

We, therefore, constructed single point mutants of endogenous AlgR that either mimic the phosphorylated (D54E) or the non-phosphorylated (D54N) state of AlgR [33,39]. These strains show comparable expression of *algR* during logarithmic growth to the WT strain and the predicted transcriptional regulation of the prepilin *fimU* cluster (S4 Fig). As expected, the phosphomimetic D54E mutant invaded cells at similar levels as the WT strain while the non-phosphorylatable D54N mutant was impaired in invasion (Fig 3C), thus phenocopying a *ΔpilA* mutant in which the PilA structural subunit of the type IV pilus is absent (Fig 3E). This confirms that phosphorylation of AlgR is required for invasion of bladder epithelial cells. The phosphomimetic D54E mutant was impaired in intracellular survival, phenocopying the *ΔalgR* strain, while the non-phophorylatable D54N mutant was able to survive intracellularly, phenocopying the WT strain (Figs 3C and 3D). Importantly, the *ΔpilA* strain was able to survive intracellularly similarly to the WT strain (Figs 3E and 3F), demonstrating that pilus expression is not necessary for intracellular survival. Single deletions of known AlgR targets known to control virulence (*algD*, *exsA*, *rhlI*, *anr*, *mucR*, *gacA*) did not phenocopy the survival defect of *ΔalgR* bacteria (S5 Fig) suggesting that the transcriptional control of intracellular survival by AlgR is complex and may involve multiple, overlapping pathways or other factors. Taken together, these results demonstrate that the phosphorylated form of AlgR is required for maximal invasion, but the non-phosphorylated form of AlgR is required for intracellular survival.

### Adaptation to the intracellular host environment is bacterial intrinsic

Bacterial adaptation to the intracellular host environment, through metabolic or other physiological changes, is necessary for intracellular bacterial survival. Previous analysis of bacterial transcriptional responses to infection had profiled a mixture of intracellular and extracellular bacteria [40–43], precluding the analysis of bacterial adaptation specifically to the intracellular environment. By using PatH-Cap, a method we developed to enrich for bacteria-derived mRNA that is particularly powerful when the fraction of bacterial mRNA is extremely low relative to total RNA in the sample [44], we defined the bacterial transcriptional changes induced by the intracellular environment. We infected cells with WT or *ΔalgR* PAO1 constitutively expressing GFP (PAO1-GFP) and performed fluorescence activated sorting (FACS) to isolate host cells that were invaded by bacteria (GFP positive). We confirmed that GFP positive cells harbor live bacteria and GFP negative cells do not by sorting cells directly onto an agar plate and determining the fraction from which bacterial colonies grew (S6 Fig). For our transcriptional analysis, we focused on an early time point of infection, 2 hpi, to understand the molecular pathways that are induced by the intracellular environment rather than the downstream consequences of intracellular bacterial survival/death. Importantly, our microscopy analysis showed that even at 3 hpi intracellular bacteria have not undergone multiple cell divisions since the majority of cells (69% for WT infected cells) still harbor only 1 or 2 bacteria. We isolated RNA from the sorted populations, prepared RNA-seq libraries using the sc-Dual-seq protocol [45] which captures both host and pathogen transcripts and applied PatH-Cap to our dual RNAseq libraries [44]. The fraction of total aligned reads that mapped to *P*. *aeruginosa* genes was 0.05–0.13% before enrichment, precluding differential expression analysis, but increased to 5.3–16.4% post-PatH-Cap (S1 Table).

**Fig 4:**
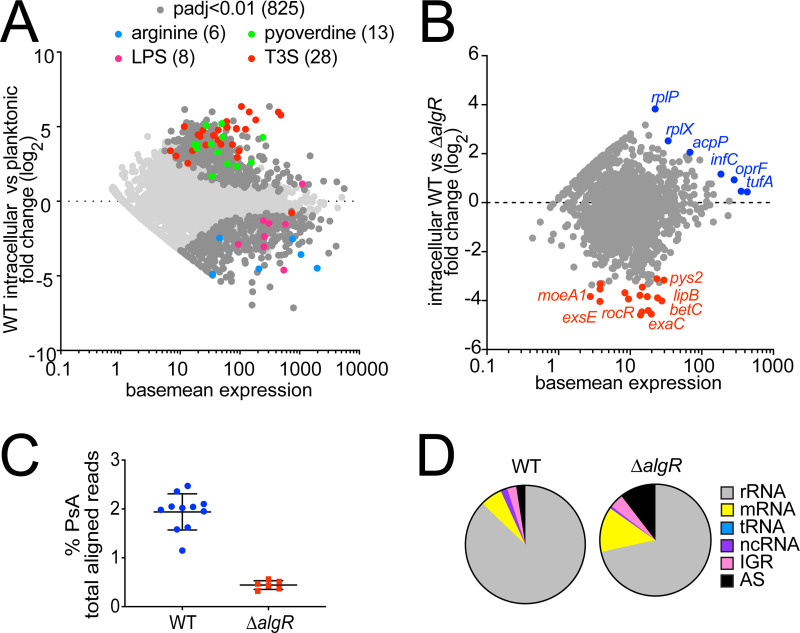
Bacterial adaptation to the host intracellular environment is rapid and is impaired in *ΔalgR* bacteria. Dual RNA-seq libraries of WT- and *ΔalgR*-invaded cells were made from cells sorted 2 hpi and enriched using *P*. *aeruginosa-*specific probes using PatH-Cap. (A) Log_2_ fold change in gene expression of intracellular versus planktonic WT bacteria. Differentially expressed genes (padj<0.01 for WT, 825 genes, dark gray) and those differentially expressed in both comparisons encoding genes belonging to pyoverdine biosynthesis (13, green), T3S secretion (28, red), arginine degradation (6, blue), and LPS biosynthesis (8, pink) GO terms are highlighted. (B) Log_2_ fold change in gene expression of intracellular *P*. *aeruginosa* WT versus *ΔalgR* bacteria. Genes highlighted in blue (7) are expressed at higher levels by intracellular WT bacteria; genes highlighted in red (17) are expressed at higher levels by intracellular *ΔalgR* bacteria (padj<0.05). Unlabeled points (10) represented uncharacterized genes that encode hypothetical proteins. (C) Percent of reads from dual RNAseq pre-PatH-Cap libraries that align to *P*. *aeruginosa* genome. (D) Composition of bacterial fraction of dual RNA-seq pre-PatH-Cap libraries.

At 2 hpi, WT bacteria demonstrated rapid and extensive transcriptional adaptation to the intracellular host environment, differentially expressing 1,268 genes (645 upregulated and 623 downregulated of 4,781 genes analyzed; padj < 0.05) compared to planktonic WT bacteria grown in LB (Fig 4A and S1 Dataset). Intracellular WT bacteria upregulated major virulence genes involved in type III secretion and biosynthesis of the siderophore pyoverdine required for iron sequestration (Fig 4A), which could be a response either to host cell contact itself and/or a response to exposure to the intracellular environment, as has been previously reported [40–43]. Interestingly, we found transcriptional downregulation of the lipopolysaccharide (LPS) O antigen biosynthesis *wpb* gene cluster (*wbpA*, *wzy*, *wbpGHI*), of arginine degradation and transport genes (*arcDABC*, *aruG, aotP*), previously shown to be upregulated in a murine acute *P*. *aeruginosa* pneumonia model [43], and of the transcriptional regulator *phoQ*, previously reported to be upregulated upon interaction with epithelial cells [46]. These transcriptional changes may reflect the restricted availability of specific nutrients in the intracellular environment resulting in a slow proliferation rate, as demonstrated by microscopy, and structural changes to the bacterial cell wall that have been shown to occur during chronic infections [47].

We then directly compared the transcriptional profile of intracellular WT and *ΔalgR* bacteria to understand differences in their ability to adapt to the intracellular environment. Surprisingly, only 24 genes were differentially expressed (padj < 0.05; 1,302 genes analyzed) (Fig 4B and S1 Dataset). The 17 genes expressed at higher levels by *ΔalgR* bacteria than WT bacteria do not appear to be enriched for any biological pathway or function, even using homology predictions on the 10 genes that encode hypothetical proteins. The seven genes expressed at higher levels by intracellular WT than *ΔalgR* bacteria consisted of five genes encoding proteins involved in translation (*tufA*, *tufB*, *infC*, *rplX* and *rplP*) and two genes (*oprF* and *acpP*) encoding a transmembrane porin and an acyl carrier protein, respectively. Apart from the translation-associated genes upregulated in WT compared to *ΔalgR* bacteria, we also noted that the composition of the *ΔalgR*-invaded libraries prior to PatH-Cap enrichment was different from WT-invaded libraries. Bacteria-derived reads composed only 0.4% of libraries made from *ΔalgR*-invaded cells compared to 1.9% of libraries made from WT-invaded cells (Fig 4C). Moreover, *ΔalgR*-invaded libraries were composed of a lower percentage of rRNA compared to WT libraries (*ΔalgR*-invaded libraries: 72% rRNA, 13% mRNA; WT-invaded libraries: 88% rRNA, 6% mRNA) (Fig 4D), a finding consistent with previous observations that under stress conditions, such as heat shock, rRNA is preferentially degraded resulting in an overall decrease in total RNA [48]. Taken together, the lower expression of translation-related genes and decreased fraction of rRNA available for translation in the *ΔalgR* strain suggests that even at this early time point, 2 hpi, *ΔalgR* bacteria may be unable to translationally adapt to the intracellular environment rapidly enough to support survival.

### Intracellular bacteria induce a host response that is predominated by activation of the NF-κB signaling pathway in non-immune cells

Host transcriptional responses, such as those downstream from TLR activation, are also critical determinants of the outcome of a bacterium invading a host cell [49]. To define the host responses during intracellular *P*. *aeruginosa* infection, we used the same model system to compare the expression profiles of cells that harbored intracellular bacteria (“invaded”, GFP positive), cells that were exposed to but were not invaded (“exposed”, GFP negative), and cells that were never exposed to bacteria (“unexposed”). We identified differentially expressed genes, analyzed them by Gene Set Enrichment Analysis (GSEA) and calculated normalized enrichment scores (NES) for Hallmark gene sets [50].

**Fig 5:**
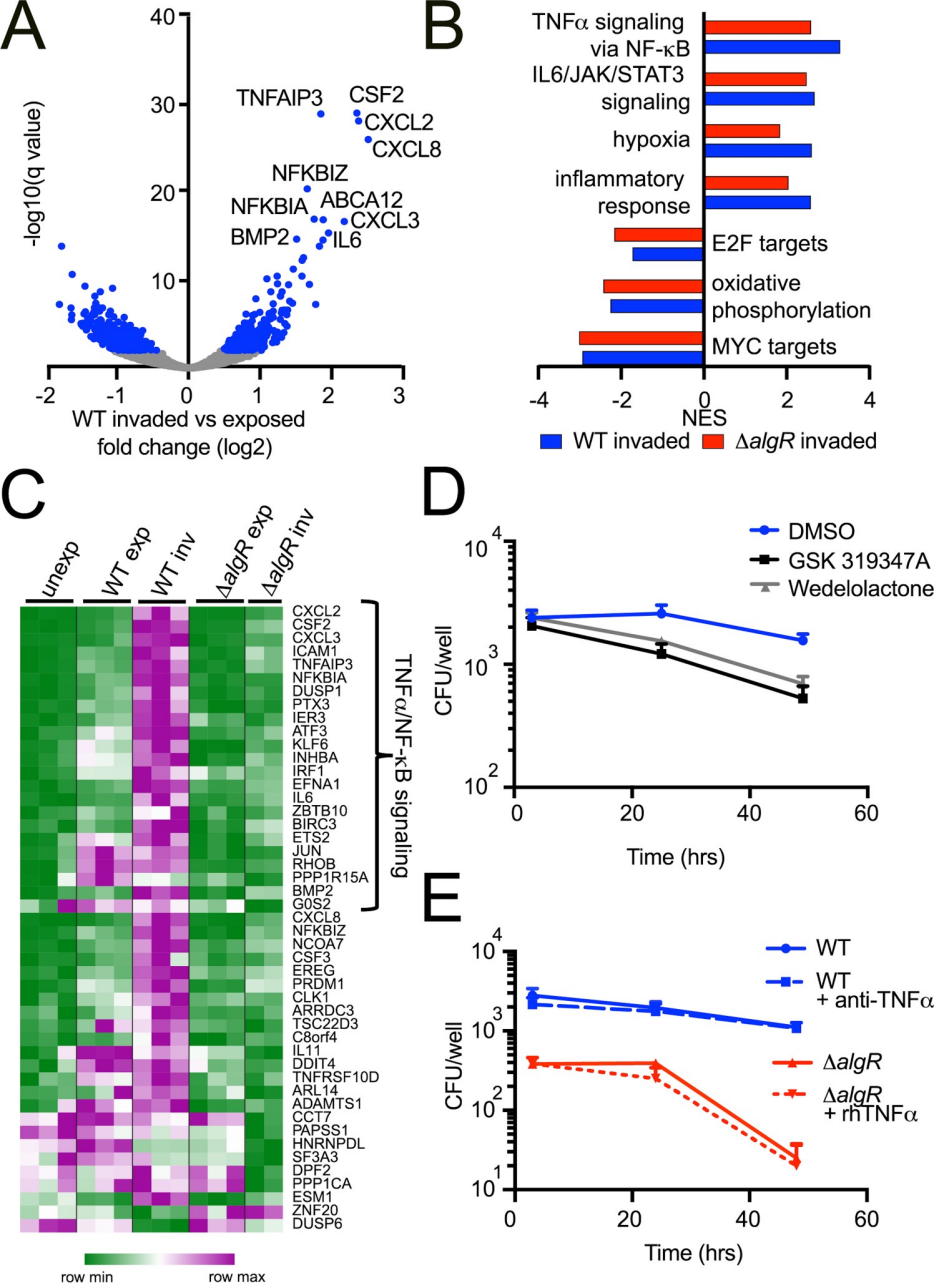
Activation of the host NF-ΚB signaling pathway is correlated with bacterial survival rather than clearance. **“**Unexposed”, “exposed” but uninfected GFP negative and “invaded” GFP positive cells were infected with WT or *ΔalgR* PAO1 GFP-expressing bacteria and sorted 2 hpi. (A) Differential expression analysis of WT exposed and invaded cells. Differentially expressed genes (padj < 0.01) highlighted in blue; top 10 genes labeled. (B) Gene expression profiles of WT- and *ΔalgR*-invaded cells were compared to unexposed cells. Normalized enrichment score (NES) for gene sets significantly enriched (FDR qval<0.005) in each comparison are shown. (C) Heat map showing genes differentially expressed between WT-invaded and *ΔalgR-*invaded cells. Genes belonging to the TNFα via NF-κB gene set are highlighted. (D) Cells were pre-treated for 30 min with GSK 319347A or Wedelolactone before infection with WT *P*. *aeruginosa* at MOI = 10. At indicated time points, the number of intracellular bacteria was determined. (E) Cells were pre-treated with TNFα blocking monoclonal antibodies or recombinant TNFα 1 hour before infection with WT or *ΔalgR P*. *aeruginosa*, respectively, at MOI = 10. At indicated time points, the number of intracellular bacteria was determined.

In both WT and *ΔalgR* -invaded cells, genes involved in TNFα signaling via NF-κB, IL6/JAK/STAT3 signaling, hypoxia and the inflammatory response were upregulated while genes categorized as MYC targets, oxidative phosphorylation and E2F targets were downregulated (FDR qval<0.005; Figs 5B and S7A). Importantly, *ΔalgR*-invaded cells do not show a transcriptional signature of apoptosis, supporting that death of host cells is not the reason for the lack of bacterial survival (S2 Dataset). Although the global transcriptional response was similar, the magnitude of the NF-κB response was lower in cells invaded by *ΔalgR* compared to WT bacteria. Of the 45 genes that were more highly expressed in WT- than *ΔalgR*-invaded cells, 23 of them were categorized as NF-κB targets including transcriptional regulators downstream from TLRs (IRF1, JUN, ATF3, ETS2), chemokines and cytokines (CXCL2, CXCL3, CXCL8, IL6, IL11, CSF3), and negative regulators of NF-κB signaling (NFKBIA, DUSP1, NFKBIZ) (Fig 5C). These results indicate that *ΔalgR*-invaded cells do not activate a unique transcriptional program that results in active bacterial clearance but, rather, these findings are consistent with our interpretation that the *ΔalgR* mutant has an intrinsic inability to adapt to the intracellular environment.

NF-κB and TNFα have long been known to be important for host defense and indeed have been shown to play a role in clearing extracellular bacteria in *P*. *aeruginosa* induced pneumonia. Yet, here, we find that stronger induction of NF-κB by WT bacteria is associated with increased intracellular survival while weaker levels of NF-κB response were associated with decreased intracellular survival of *ΔalgR* bacteria. To explore the possibility that the stronger NF-κB response to WT bacteria could paradoxically be contributing to enhanced intracellular bacterial survival relative to *ΔalgR* bacteria, we blocked NF-κB signaling with two IKK inhibitors, GSK 319347A and Wedelolactone [51,52], and also tested wortmannin, a PI3K inhibitor that has been shown to block NF-κB activation by LPS but not TNFα [53]. Pre-treatment with IKK inhibitors or wortmannin all reduced intracellular survival of WT bacteria (Figs 5D and S7B), thus demonstrating that NF-κB signaling is necessary for intracellular bacterial survival. However, activation of the NF-κB pathway with exogenous rTNFα did not enhance the intracellular survival of *ΔalgR* bacteria nor did blockade of TNFα with antibodies result in reduced intracellular survival of WT bacteria (Fig 5E) even though both TNFα and its receptors, TNFRSF1A and TNFRSF1B, are expressed during infection (S7C Fig), suggesting that other signaling pathways activated during infection may also be involved. Thus, activation of NF-κB signaling in host cells appears to be necessary, but not sufficient, for intracellular bacterial survival.

### Bacterial intracellular survival increases in vivo persistence in a mouse model of urinary tract infection

**Fig 6:**
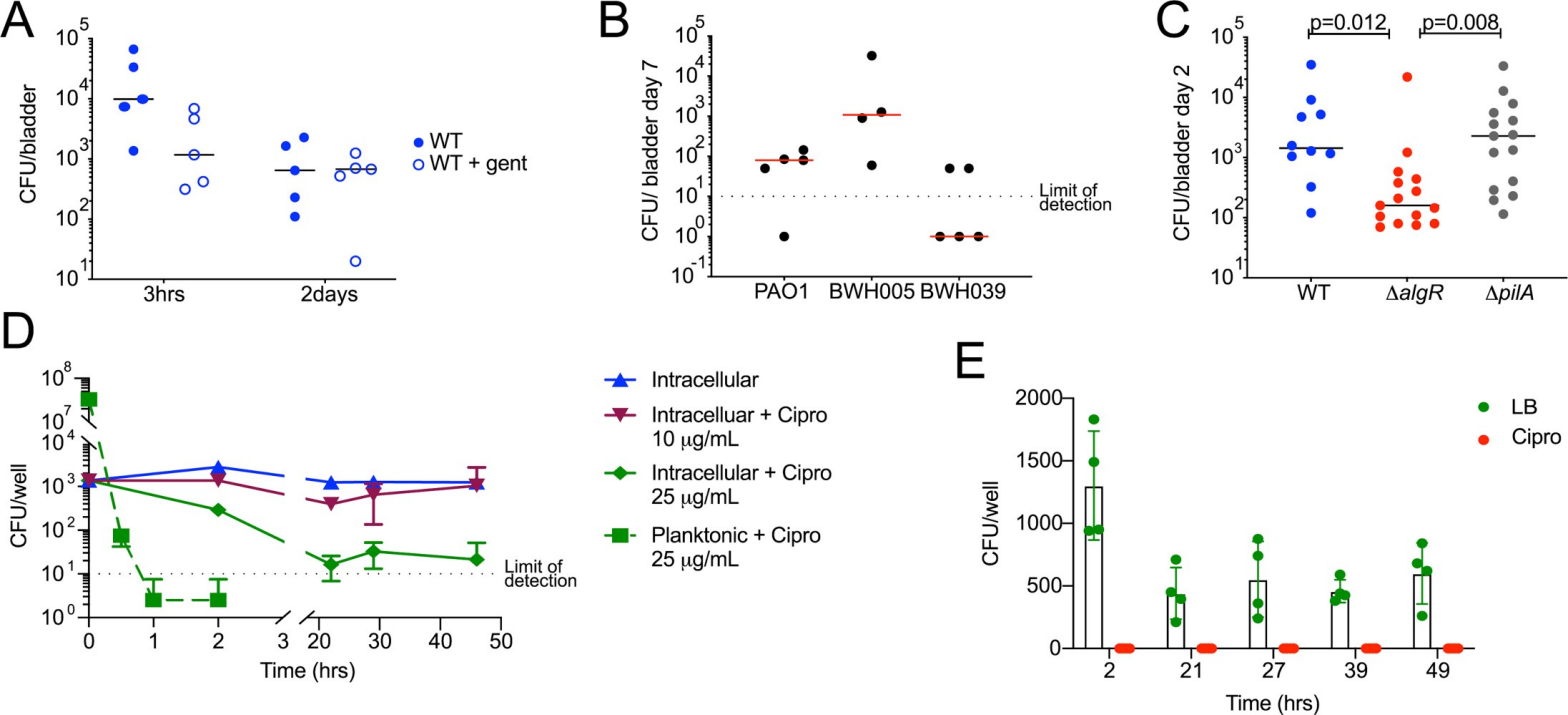
Bacterial intracellular survival increases *in vivo* persistence in a mouse model of urinary tract infection and decreases antibiotic efficacy. (A-C) C57BL/6 female mice were transurethrally inoculated with 1–6 x 10^7^ CFU *P*. *aeruginosa*. (A) At 3 hours or 2 days post-infection, bladders were harvested and incubated for 30 minutes in media alone or gentamicin to kill extracellular bacteria; the entire bladder was then homogenized to determine total (untreated) and intracellular (gentamicin treated) bacterial load. n = 5 per time point per condition. (B) Mice were infected with the lab-adapted strain PAO1 or two clinical strains isolated from urine (BWH005 and BWH039). Seven days post-infection, bladders were harvested, homogenized and bacterial load was determined. n = 4 or 5 per strain. (C) Mice were infected with WT, *ΔalgR* or *ΔpilA* PAO1 *P*. *aeruginosa*. Two days post-infection, bladders were harvested and homogenized to determine bacterial load. Adjusted P value of Dunn’s multiple comparisons test between groups displayed. n = 10–15 mice from 3 independent experiments. (D) Bladder epithelial cells were infected with WT bacteria for 2 hours and then incubated with gentamicin plus the cell permeable antibiotic ciprofloxacin at either 10 or 25 μg/mL. At the indicated time points intracellular bacterial load was determined. For comparison, the same inoculum of bacteria was treated with 25 μg/mL ciprofloxacin without host cells and the number of surviving bacteria was determined. (E) Cells were infected with WT bacteria and treated as in D with ciprofloxacin (10 μg/mL) and the lysate was plated in the absence of presence of ciprofloxacin (10 μg/mL). Minimum level of detection was 5 CFU/well.

To determine the *in vivo* significance of intracellular survival, we compared survival of WT versus *ΔalgR* bacteria in a mouse model of urinary tract infection [54]. Infection with UPEC in this model results in intracellular survival of bacteria within the bladder epithelium; some of these cells are shed in the first few hours after infection exposing deeper layers of cells that also become reservoirs of intracellular bacteria [54]. We infected mice transurethrally with *P*. *aeruginosa*, harvested bladders at the indicated time points, and plated the homogenates to determine bacterial load. To distinguish between intra- and extracellular bacteria, we incubated explanted bladders in the presence or absence of gentamicin prior to homogenization following an established *ex vivo* gentamicin protection assay protocol [55]. At 3 hpi, 90% of WT bacteria were killed by *ex vivo* exposure to gentamicin, demonstrating that only 10% of bacteria were intracellular at this time point. By 2 days post-infection (dpi), however, the majority of WT bacteria were intracellular (Fig 6A) and able to persist out to 7 days in 4 out of 5 mice (Fig 6B). This persistence at 7 dpi was also was also true for two clinical strain isolated from the urine of patients, with one strain persisting in all 4 mice infected and the other one persisting in 2 out of 5 mice (Fig 6B). Importantly, infection with *ΔalgR* bacteria resulted in a lower intracellular bacterial load recovered from the bladder at 2 dpi, compared to WT bacteria. This decrease cannot be attributed to a decrease in pilus-dependent invasion since *ΔpilA* bacteria were able to survive within the bladder to the same extent as WT bacteria (Fig 6C), just as they were able to survive intracellularly in our *in vitro* model. These data demonstrate that the *in vitro* model recapitulates *in vivo* infection, and that intracellular survival contributes to bladder colonization in murine urinary tract infection.

### Bacterial intracellular infection decreases antibiotic efficacy

Finally, in the context of various bacterial infections, a fraction of intracellular bacteria has been shown to become antibiotic tolerant [12,56,57]. To determine whether intracellular *P*. *aeruginosa* form a similar population within bladder epithelial cells, we treated infected cells with a concentration of the cell permeable antibiotic ciprofloxacin 10–25 times above the minimum inhibitory concentration that rapidly sterilizes the bacteria in culture. Indeed, we observed a biphasic kill curve, particularly with the higher concentration of antibiotic, that results in a stable population of antibiotic tolerant intracellular bacteria that are resistant to killing 48 hours after treatment (Fig 6D). Importantly, these intracellular bacteria have not acquired ciprofloxacin resistance, as they are sensitive to the antibiotic after isolation (Fig 6E).

## Discussion

Our understanding of *P*. *aeruginosa* infection has been predominantly focused on its ability to form extracellular biofilms, or to invade and kill host cells. Here, we demonstrate that *P*. *aeruginosa* also has the ability to survive intracellularly within human bladder epithelial cells and this survival potentially contributes to the formation of a persistent intracellular reservoir in a mouse model of urinary tract infection. Using dual transcriptional analysis, we define the host and bacterial adaptations to *P*. *aeruginosa* intracellular survival within bladder epithelial cells. Moreover, this intracellular population is antibiotic tolerant and, therefore, may serve as a reservoir that could give rise to antibiotic resistant bacteria, further complicating the treatment of these infections [1].

*Pseudomonas aeruginosa* is an important uropathogen in both catheter and non-catheter associated UTIs [8,13]. It is most prevalent in catheter associated UTIs where it is correlated with high mortality in hospitalized patients [58]. Interestingly, it has been shown that *P*. *aeruginosa* UTIs result in higher readmission rates compared to UTIs caused by other uropathogens, underscoring the clinical importance of this problem [9]. Bacteriuria has been shown to persist in patients even after catheter removal and, although antibiotic prophylaxis prior to removal of the catheter shows some benefit in preventing subsequent UTIs, not all patients respond to this treatment [59]. We speculate that intracellular survival of bacteria within host tissues during catheter usage may impede response to antibiotic treatment after catheter removal, since our data demonstrate that intracellular bacteria adopt an antibiotic tolerant state. It will be of interest to assess whether intracellular *P*. *aeruginosa* can indeed be detected in patients who undergo catheterization or suffer from chronic UTIs. Such clinical data would support the notion that bacterial adaptation to the intracellular niche promotes pathogenesis, as has been shown for UPEC in persistent UTIs [60]. Host colonization by uropathogens occurs in the lumen of the bladder where the stratified epithelium is composed of an undifferentiated basal layer, followed by 3–4 layers of intermediate and then highly differentiated superficial umbrella cells [54]. The cell line used for these studies does not express umbrella cell markers, such as uroplakin, and therefore most closely resembles undifferentiated urothelial cells [61]. It has been shown that invasion of superficial cells by UPEC results in rapid bacterial replication and formation of intracellular bacterial communities (IBCs). This superficial layer of invaded cells is exfoliated within 24 hours after infection, which serves as a host defense mechanism and reduces bacterial load [61]. Critically, exfoliation exposes the deeper layers of the urothelium which may in turn be colonized; it is within these underlying epithelial layers that uropathogens may persist in a quiescent state [62], which we propose is the case in our model. Indeed, UPEC has been shown to remain viable for months in murine models of UTI, presumably within these deeper layers of the urothelium, and giving rise to quiescent intracellular reservoirs (QIRs) [54]. Here, we demonstrate that *P*. *aeruginosa* is able to invade and persist within the murine bladder epithelium with similar kinetics as UPEC, showing a decrease in bacterial load over the course of 7 days of infection [54]. QIRs are more prevalent in Balb/c compared to C57BL/6 mice demonstrating host variability in the resolution of UTIs [61]. General host-specific responses to infection, as well as pathogen-specific ones, remain to be identified and will undoubtably further our understanding of this complex interaction. Here, we additionally show that there is significant variability in the ability of clinical isolates from urine to persist in this mouse model: one clinical isolate, BWH005, persisted for 7 days in all mice infected while the other one, BWH039, persisted only in 2 out of 5 mice infected. These differences may be due to either stronger host responses or other bacterial-intrinsic defects not related to intracellular survival since the BWH039 strain was able to survive intracellularly in our *in vitro* model.

Previous simultaneous transcriptional analyses of *P*. *aeruginosa* and host cells either *in vivo* or *in vitro* have profiled a mixture of intracellular and extracellular bacteria as well as a mixture of exposed and invaded cells [40–43]. Our ability to exclusively profile intracellular bacteria and to differentiate between the host response to exposure versus invasion advances our understanding of this complex host-pathogen interaction. For example, downregulation of LPS biosynthesis in our model suggests that modification of LPS may be specifically required for adaption to the intracellular environment. Meanwhile, host transcriptional profiling demonstrated that intracellular bacteria are sensed by host cells in a response that is dominated by activation of NF-κB signaling, likely downstream from TLRs. Comparing invasion by WT bacteria to invasion by a mutant that is defective for intracellular survival due to disruption of the global regulator of virulence AlgR, we found that the host NF-κB response is stronger in WT-invaded compared to *ΔalgR*-invaded cells. A recent study showed that high and sustained NF-κB signaling and maximal JNK signaling only occurred in response to live bacteria that were sensed by the host as a threat, as opposed to heat-killed bacteria [63], suggesting that the relatively muted NF-κB signaling on the part of the host in response to *ΔalgR* invasion may be because it is not sensing the same threat level as it does from WT bacteria. Paradoxically, while the NF-κB response plays a role in clearing of extracellular bacteria [17], it appears to be required for intracellular bacterial survival, as its inhibition with IKK inhibitors leads to decreased survival of intracellular WT bacteria. However, it is not itself sufficient for intracellular bacterial survival, because its activation with exogenous TNFα did not rescue the intracellular survival of the *ΔalgR* mutant. This NF-κB host response thus appears to promote the maintenance of an intracellular bacterial reservoir during persistent infection. These divergent roles of NF-κB may be due to differences between professional innate immune cells and epithelial cells, with *P*. *aeruginosa* being readily cleared from macrophage-like cells but surviving inside epithelial cells. In support of this dual role for NF-κB signaling, more severe bladder inflammation is seen in mice infected with UPEC that go on to develop chronic cystitis and persistent bacteriuria: the establishment of intracellular bacterial reservoirs is correlated with higher levels of IL-5, IL-6, CSF and the IL-8 homolog KC [64]. As in our model, however, it is unclear whether the exacerbated host response is the cause or the consequence of the chronic infection.

This analysis revealed that there is heterogeneity in the outcome of infection of the small fraction (0.5%) of epithelial cells that are initially invaded by *P*. *aeruginosa*. With WT bacteria, we observed two distinct outcomes of infection: bacterial replication with an intracellular replication rate that is much slower than is seen in culture or for other intracellular pathogens, and bacterial survival or persistence without proliferation, comprising 4% of invaded cells at 48 hpi. A third outcome, the inability to survive intracellularly, was exemplified by *ΔalgR* bacteria, but is likely to also occur with a fraction of WT bacteria. Determining both the host and bacterial molecular pathways governing these outcomes will likely require analysis at the single cell level since bulk analysis, as presented here, averages the transcriptional responses of this heterogenous intracellular population. Although our analysis identified genes differentially expressed between intracellular WT and *ΔalgR* bacteria, it was restricted to genes detected in both strains (1,302 genes). Genes with no detectable expression were excluded from our analysis due to the inability to differentiate between true lack of expression and lack of technical detection. As such, genes required for intracellular survival that were not upregulated by *ΔalgR* bacteria, and therefore not detected in our RNAseq libraries, were not identified by our transcriptional analysis. Thus, it is possible that more genes are in fact differentially regulated between these two strains that may help explain the difference in their ability to survive intracellularly. Technical improvements in capture and detection of lowly expressed transcripts, which in these dual host-bacteria RNA-seq libraries comprise all bacterial transcripts, will increase the sensitivity of detection of the bacterial transcriptome and enable more comprehensive analysis of all genes [44].

Persistent bacterial infections that are resistant to antimicrobial therapy pose a serious and growing public health problem, yet novel approaches to addressing these infections are lacking. We have demonstrated that the previously unappreciated ability of *P*. *aeruginosa* to survive intracellularly may play an important role in contributing to the chronicity and recurrence of urinary tract and, potentially, other infections caused by *P*. *aeruginosa*. Finding novel approaches to eradicating this intracellular population, potentially by targeting host pathways such as NF-κB signaling, could transform our ability to manage chronic and/or recurrent infections such as UTIs.

## Methods

### Ethics statement

*Pseudomonas aeruginosa* clinical strains were collected by the Clinical Microbiology Laboratory at Brigham and Women’s Hospital (BWH). Collection of strains was approved by Partners Healthcare Internal Review Board (2012P001062) with waiver of patient consent, since bacterial isolates were obtained from discarded, de-identified material from the clinical laboratories. All vertebrate animal experiments were done with the approval of the Massachusetts General Hospital’s Institutional Animal Care and Use Committee (2007N000153).

### Strain selection and construction

Mutants with in-frame nonpolar deletions tested to identify bacterial pathways required for intracellular survival of *P*. *aeruginosa* were *ΔalgR*, *ΔexsA*, *ΔphzA1/2*, *ΔpqsA*, *ΔpqsE* and *ΔmvfR* [65]. BWH001, BWH05, BWH015, BWH039, and BWH060 clinical strains are all *exoS*^+^, *exoU*^-^, *exlA*^-^. *P*. *aeruginosa* PAO1-GFP was constructed by integrating a single copy of the pGFPmut3.1 gene driven by a constitutive insulated promoter [66] into the bacterial chromosome using the mini-Tn7 vector [67]. Gene deletions in PAO1 were performed by allelic exchange as previously described [68] and were confirmed by PCR amplification and sequencing. For complementation, the *algR* gene was cloned into pHERD20T using complement forward and reverse primers; expression is driven by the pBAD promoter in this plasmid. For site directed mutagenesis, the *algR* gene was first cloned into the pEXG2-GW vector using replace forward and reverse primers; site directed mutagenesis was performed using the Q5 Site-Directed Mutagenesis Kit (NEB) using the SDM primers. Mutagenesis was confirmed by sequencing. The mutagenized versions were then used to replace the endogenous *algR* locus by allelic exchange. S2 Table includes all primers used for in-frame deletions, *algR* complementation and site-directed mutagenesis.

### Gentamicin protection assay

5637 human bladder epithelial cells (RRID: CVCL_0126), A549 human lung epithelial cells (RRID: CVCL_0023) or macrophage-like murine J774 cells (RRID: CVCL_4692) were seeded overnight in RPMI media supplemented with 10% FBS. Log-phase bacteria grown in LB (OD_600_ = ~0.3) and diluted in media were used to infect cells at the indicated MOI and centrifuged to synchronize infection. Cells were incubated for 1–2 hours, extracellular bacteria were removed, media containing 200 μg/ml gentamicin was added and incubated for an additional 1–2 hours. Media was then replaced with media containing 25 μg/ml gentamicin, to prevent reinfection, until cells were harvested. For enumeration of intracellular bacteria, at the indicated time points, cells were washed with PBS and lysed in 1 mL of PBS + 0.1% Triton X-100 and plated to LB agar + 15 μg/mL irgasan to enumerate CFU. For enumeration of adherent bacteria, cells were incubated in the absence of gentamicin for 3 hours, washed with PBS and harvested as described at 3 hpi. Viability of host cells was measured using CellTiter-Glo (Promega) per the manufacturer’s protocol; data shown is relative to uninfected cells harvested at the same time point to control for proliferation of this cell line.

Where indicated, cells were pre-incubated with recombinant human TNFα protein (125 pg/mL; R&D Systems), anti-human TNFα antibodies (0.1 μg/mL; RRID:AB_2203945), IKK inhibitor GSK 319347A (1 μM; Tocris), Wedelolactone CAS 524-12-9 (10 mM; Calbiochem) or Wortmannin CAS 19545-26-7 (100 nM; Sigma-Aldrich) for 30–60 minutes before infection and throughout all incubations. Ciprofloxacin was used at a concentration of 10 or 25 μg/ml as indicated starting at 3 hpi. For the confirmation of ciprofloxacin sensitivity, cell lysates were plated on plates without antibiotic (LB) or containing 10 μg/ml ciprofloxacin.

### Flow cytometry

Fluorescently activated cell sorting was performed using a BD FACSAria II (BD Biosciences). Cells were infected as described, incubated for 1hr before the addition of gentamicin and detached using trypsin 2 hpi. DAPI was added prior to sorting to discriminate dead/dying cells from healthy cells. GFP+ and GFP- cells were sorted using gates analogous to the “high” and “neg” gates, respectively, shown in S6 Fig. For transcriptional analysis, populations of cells were sorted directly into lysis buffer (RNA-Gem Lysis buffer, 1% BME and RNAse inhibitor) and frozen. For confirmation of viability of intracellular bacteria, single cells were sorted directly onto agar plates, the plates were incubated overnight at 37°C and the number of colonies that grew out were counted.

### Microscopy analysis

Cells were seeded on glass coverslips, infected at MOI = 10, fixed with 1% formaldehyde, stained with anti-LAMP-1 antibodies (RRID: AB_775978) and DAPI, imaged using a Zeiss LMS800 confocal microscope and acquired and analyzed using the Zeiss ZEN software. At least 50 invaded cells per condition were chosen randomly from 4–6 biological replicates. Z-series were collected that captured the entirety of the cell. Maximum Intensity Projections of the XY, XZ and YZ planes are shown. Intracellular bacterial load was determined manually. Intracellular bacterial were considered to be LAMP-1 associated when they were at least 50% surrounded by LAMP-1 staining.

### RNA isolation and library construction of planktonic bacteria

Bacteria were grown to mid-logarithmic phase (OD_600_ = ~0.3) in LB, harvested by centrifugation, resuspended in TRIzol (ThermoFisher Scientific), incubated for 10 minutes at room temperature, transferred to tubes with zirconia beads, and bead beat three times for 60 seconds each, with 60 seconds incubations on ice in between pulses. RNA was extracted using the Directzol RNA extraction kit (Zymo Research). Libraries were generated using the RNAtag-Seq protocol [69].

### Library construction

For host transcriptional analysis, 5,000 cells were sorted per sample (three independent biological infections). Samples were lysed by incubating at 75°C for 5 minutes and RNA was isolated using SPRI paramagnetic beads. rRNA was depleted using RiboGone-Mammalian (Clontech Laboratories, Inc), cDNA was synthesized using the SMARTer Universal Low Input RNA Kit (Clontech Laboratories, Inc) and library preparation was performed using the Low Input Library Prep Kit v2 (Clontech Laboratories, Inc).

For bacterial intracellular transcriptional analysis, populations of 1,000 cells were sorted (for WT-invaded: four technical replicates from each of three independent biological infections; for *ΔalgR* -invaded: two technical replicates from each of three independent biological infections). Samples were lysed by incubating at 75°C for 5 minutes. Dual RNA-seq libraries were made following the sc-Dual-seq protocol [45]. *P*. *aeruginosa* mRNA transcripts were enriched with PatH-Cap as previously described [44]. Briefly, dual RNA-seq libraries composed of host and bacterial transcripts were incubated with biotinylated probes specific for the PAO1 transcriptome. The targets derived from bacterial mRNA were then pulled down by their corresponding biotinylated probe using streptavidin-coated beads, PCR amplified and sequenced.

### Sequencing, alignment and analysis

Paired-end sequencing of RNA-seq libraries was performed on Illumina NovaSeq and NextSeq platforms at the Broad Institute Genomics Core. Reads were aligned to the PAO1 genome from Refseq (NC_002156) using BWA and the human transcriptome generated from Ensembl gene annotations (GRCh38/hg38) using BBMap as previously described [44]. For RNAtag-Seq and SMARTer libraries both read1 and read2 were aligned; for Dual-seq libraries only read2 was aligned after trimming reads with stretches of 7 or more A’s. An in-house script was used for enumeration and metrics generation. For SMARTer libraries, reads aligned to sense and antisense genes were summed since this library construction protocol is not stranded. For Dual-Seq libraries, transcripts with the same Unique Molecular Identifier (UMI) were collapse into a single read using an in-house script by clustering with respect to UMI as previously described [44]. All gene expression analysis of intracellular bacteria was done after duplicate reads were removed.

For analysis of Dual-seq libraries, libraries were filtered based on host alignment of pre-PatH-Cap libraries: percent protein coding regions < 45%; percent sense protein coding regions > 65%; unique host transcripts (UMI collapsed) in protein coding regions >3,000. For analysis of bacterial transcriptome, technical replicates were summed, and only genes detected (>0 counts) in at least two of three biological replicates of intracellular bacteria for each strain were analyzed. Differential expression analysis was conducted with DESeq2 [70]. Identified DEGs were analyzed using the PANTHER Classification System [71] using an overrepresentation test (Released 20181113) in the GO ontology database (Released 2018-11-15).

For analysis of host transcriptome, only genes detected with ≥10 reads were included. Differential expression analysis was conducted with DESeq2 [70]. Identified DEGs were then analyzed by Gene Set Enrichment Analysis (GSEA) using the Molecular Signatures Database (MSigDB) Hallmark gene set collection v 6.2 [50,72]. Normalized enrichment scores (NES) were calculated in MSigDB.

### Murine urinary tract infections

We adapted the established murine UPEC urinary tract infection model to the use of *P*. *aeruginosa* [54]. Female 12–16 week old C57BL/6 mice from Jackson Laboratories were used. Bacteria were grown in LB to stationary phase, collected by centrifugation, washed in PBS, and resuspended in PBS. 1–6 x 10^7^ bacteria in 50 μl were instilled in the bladder through a 0.61/0.28 mm (inner/outer diameter) catheter while animals were anesthetized by inhaled isofluorane. At the indicated times post infection, mice were euthanized per protocol by CO_2_ overdose and cervical dislocation, bladders were homogenized in 1 mL of PBS + 0.1% Triton X-100 and plated to LB agar + 15 μg/mL irgasan to enumerate the bacterial burden. The *ex vivo* gentamicin protection assay has been previously described [55]. Briefly, bladders were washed in PBS 3 times and incubated in the presence or absence of 200 μg/mL gentamicin for 30 min at room temperature. After this incubation, bladders were washed and homogenized and lysates were plated to enumerate bacterial burden.

### Statistical analysis

GraphPad Prism software (GraphPad Software) was used for the statistical analysis and graph preparation.

## Supporting information

S1 Fig Gentamicin prevents growth of *P. aeruginosa* in RPMI. PAO1 was incubated in RPMI + 10% FBS with increasing concentration of gentamicin. OD_600_ was measured at 16 hours.
(TIF)

S2 Fig Intracellular survival of *P. aeruginosa* extends to clinical strains and other host cell types. (A-B) Cells were infected with clinical strains isolated from urine at MOI = 1. (A) Host cell survival was measured 24 hpi relative to uninfected cells harvested at the same time point. (B) Percentage of intracellular bacteria recovered at 48 hpi relative to 3 hpi. Each data point represents an independent experiment. (C) A549 lung epithelial cells were infected with WT PAO1. Number of surviving intracellular bacteria were determined at 24 and 48 hpi. Average of two independent experiments is shown. (D) The macrophage-like cell line J774 was infected with WT PAO1. Number of surviving intracellular bacteria were determined at 24 and 48 hpi. Data shown is representative of three independent experiments.
(TIF)

S3 Fig *ΔalgR* strain does not have a general fitness defect. (A, B) Growth of WT and *ΔalgR* was followed at 37°C in either LB (A) or M9 minimal medium (B) by measuring OD_600_. (C) Antibiotic susceptibility to gentamicin (GM), levofloxacin (LVX), azithromycin (AZM), amikacin (AN), colistin (CL), tobramycin (NN), ciprofloxacin (CIP), imipenem (IMP) and ceftazidime (CAZ) was determined by disc diffusion. Average zone of inhibition for three discs is shown. (D) Survival of WT and *ΔalgR* after exposure to 25μM NaOCl in PBS + 10mM glucose determined by plating for CFU.
(TIF)

S4 Fig Expression of *algR* and *fimUpilVWXY1Y2E* operon during log phase by RNAseq. RNA-seq libraries were made of WT, *ΔalgR*, *ΔalgZ* and D54N and D54E *algR* point mutants grown in LB during logarithmic phase. Transcripts per million (TMPs) for (A) *algR* gene and (B) the sum of the genes in the *fimU* operon (*fimUpilVWXY1Y2E)*.
(TIF)

S5 Fig Single deletions of known AlgR targets and/or pathways do not phenocopy intracellular survival defect. In-frame nonpolar deletions of known AlgR targets were made in the PAO1 background. Cells were infected and harvested at the indicated time points. (A) Quorum sensing regulator *lasR* and inducers *lasI* and *rhlI*. Alginate producing enzyme *algD*. (B) Transcriptional activator of anaerobic gene expression *anr* and two-component regulator controlling quorum sensing *gacD*. (C) Type III secretion regulator *exsA* and needle component *pscC*. (D) Alginate biosynthesis regulator *mucR*.
(TIF)

S6 Fig GFP positive cells harbor live bacteria while GFP negative cells do not. Cells were infected at various MOIs with the GFP expressing WT strain. At 4 hpi cells were harvested and single cells were sorted directly onto agar plates. (A) Gating scheme based on side scatter (SSC) and GFP fluorescence. (B) Plates were incubated overnight and the number of colonies, relative to the number of single cells sorted was determined. For MOI = 50 and 25, 162 cells were sorted per gate. For MOI = 5, 81 cells were sorted per gate. Representative of two independent experiments.
(TIF)

S7 Fig Activation of the host NF-ΚB signaling pathway is correlated with bacterial survival rather than clearance. (A) Log_2_ fold change gene expression of WT-invaded and *ΔalgR*-invaded cells compared to unexposed cells. Genes differentially expressed between WT-invaded and *ΔalgR*-invaded cells highlighted in red. (B) Cells were pre-treated for 60 min with wortmannin before infection with WT *P*. *aeruginosa* at MOI = 10. At indicated time points the number of intracellular bacteria was determined. (C) Transcripts per million (TMPs) for genes encoding TNFα and its receptors, TNFRSF1A and TNFRSF1B, from the indicated sorted populations. Each data point represents a biological replicate.
(TIF)

S1 Table PatH-Cap enrichment enables analysis of *P. aeruginosa* invaded cells. Dual RNAseq libraries were made with the scDualseq protocol and were sequenced before and after enrichment of *P*. *aeruginosa* mRNA derived transcripts with PatH-Cap. Percentage of total aligned reads that mapped to the *P*. *aeruginosa* genome and specifically to *P*. *aeruginosa* mRNA are shown. *of total aligned reads (host + bacteria aligned).
(DOCX)

S2 Table Oligonucleotides used. Primers used for in-frame nonpolar deletions, *algR* complementation and site-directed mutagenesis.
(DOCX)

S1 Dataset Bacterial transcriptional analysis of *P. aeruginosa* infection. Invaded GFP positive (“inv”) cells were sorted 2 hours post-infection with WT or *ΔalgR* PAO1 GFP-expressing bacteria. RNAseq libraries were made using the scDualseq protocol and enriched for *P*. *aeruginosa* mRNA using PatH-Cap. Libraries from log phase planktonic culture grown in LB (“log”) were made using the RNAtag-Seq protocol. Raw counts, and DESeq2 analysis (log_2_ fold change and pajd) shown for intracellular WT vs intracellular *ΔalgR* bacteria and intracellular vs log phase bacteria.
(XLSX)

S2 Dataset Host transcriptional analysis of P. aeruginosa infection. Unexposed (“unexp”), exposed but uninfected GFP negative (“exp”) and invaded GFP positive (“inv”) cells were sorted 2 hours post-infection with WT or *ΔalgR* PAO1 GFP-expressing bacteria. RNAseq libraries were made using the SMARTer protocol. Raw counts and DESeq2 analysis (log_2_ fold change and pajd) shown for various comparisons.
(XLSX)

## References

[1] Fauvart M, De Groote VN, Michiels J. Role of persister cells in chronic infections: clinical relevance and perspectives on anti-persister therapies. Journal of medical microbiology. 2011;60(Pt 6):699–709. 10.1099/jmm.0.030932-0 21459912

[2] Foxman B. Epidemiology of urinary tract infections: incidence, morbidity, and economic costs. Dis Mon. 2003;49(2):53–70. 10.1067/mda.2003.7 12601337

[3] Anderson GG, Palermo JJ, Schilling JD, Roth R, Heuser J, Hultgren SJ. Intracellular bacterial biofilm-like pods in urinary tract infections. Science. 2003;301(5629):105–7. 10.1126/science.1084550 12843396

[4] Hunstad DA, Justice SS. Intracellular lifestyles and immune evasion strategies of uropathogenic Escherichia coli. Annual review of microbiology. 2010;64:203–21. 10.1146/annurev.micro.112408.134258 20825346

[5] Mysorekar IU, Hultgren SJ. Mechanisms of uropathogenic Escherichia coli persistence and eradication from the urinary tract. Proceedings of the National Academy of Sciences of the United States of America. 2006;103(38):14170–5. 10.1073/pnas.0602136103 16968784 PMC1564066

[6] Lewis AJ, Richards AC, Mulvey MA. Invasion of Host Cells and Tissues by Uropathogenic Bacteria. Microbiol Spectr. 2016;4(6). 10.1128/microbiolspec.UTI-0026-2016 28087946 PMC5244466

[7] Gaynes R, Edwards JR, National Nosocomial Infections Surveillance S. Overview of nosocomial infections caused by gram-negative bacilli. Clin Infect Dis. 2005;41(6):848–54. 10.1086/432803 16107985

[8] Marcus N, Ashkenazi S, Samra Z, Cohen A, Livni G. Community-acquired Pseudomonas aeruginosa urinary tract infections in children hospitalized in a tertiary center: relative frequency, risk factors, antimicrobial resistance and treatment. Infection. 2008;36(5):421–6. 10.1007/s15010-008-7328-4 18795227

[9] Gomila A, Carratala J, Eliakim-Raz N, Shaw E, Wiegand I, Vallejo-Torres L, et al. Risk factors and prognosis of complicated urinary tract infections caused by Pseudomonas aeruginosa in hospitalized patients: a retrospective multicenter cohort study. Infect Drug Resist. 2018;11:2571–81. 10.2147/IDR.S185753 30588040 PMC6302800

[10] Fleiszig SM, Zaidi TS, Fletcher EL, Preston MJ, Pier GB. Pseudomonas aeruginosa invades corneal epithelial cells during experimental infection. Infection and immunity. 1994;62(8):3485–93. 10.1128/IAI.62.8.3485-3493.1994 8039920 PMC302982

[11] Junkins RD, Shen A, Rosen K, McCormick C, Lin TJ. Autophagy Enhances Bacterial Clearance during P. aeruginosa Lung Infection. PloS one. 2013;8(8):e72263. 10.1371/journal.pone.0072263 24015228 PMC3756076

[12] Garcia-Medina R, Dunne WM, Singh PK, Brody SL. Pseudomonas aeruginosa acquires biofilm-like properties within airway epithelial cells. Infection and immunity. 2005;73(12):8298–305. 10.1128/IAI.73.12.8298-8305.2005 16299327 PMC1307054

[13] Newman JW, Floyd RV, Fothergill JL. The contribution of Pseudomonas aeruginosa virulence factors and host factors in the establishment of urinary tract infections. FEMS microbiology letters. 2017;364(15). 10.1093/femsle/fnx124 28605563

[14] Hauser AR. The type III secretion system of Pseudomonas aeruginosa: infection by injection. Nature reviews Microbiology. 2009;7(9):654–65. 10.1038/nrmicro2199 19680249 PMC2766515

[15] Cornelis P, Dingemans J. Pseudomonas aeruginosa adapts its iron uptake strategies in function of the type of infections. Front Cell Infect Microbiol. 2013;3:75. 10.3389/fcimb.2013.00075 24294593 PMC3827675

[16] Lavoie EG, Wangdi T, Kazmierczak BI. Innate immune responses to Pseudomonas aeruginosa infection. Microbes Infect. 2011;13(14–15):1133–45. 10.1016/j.micinf.2011.07.011 21839853 PMC3221798

[17] Sadikot RT, Zeng H, Joo M, Everhart MB, Sherrill TP, Li B, et al. Targeted immunomodulation of the NF-kappaB pathway in airway epithelium impacts host defense against Pseudomonas aeruginosa. J Immunol. 2006;176(8):4923–30. 10.4049/jimmunol.176.8.4923 16585588

[18] Engel J. Pseudomonas aeruginosa internalization by non-phagocytic cells. In: Ramos JL, Filloux A, editors. Pseudomonas: Springer; 2007. p. 343–68.

[19] Apodaca G, Bomsel M, Lindstedt R, Engel J, Frank D, Mostov KE, et al. Characterization of Pseudomonas aeruginosa-induced MDCK cell injury: glycosylation-defective host cells are resistant to bacterial killing. Infection and immunity. 1995;63(4):1541–51. 10.1128/IAI.63.4.1541-1551.1995 7890421 PMC173187

[20] Chandler CE, Horspool AM, Hill PJ, Wozniak DJ, Schertzer JW, Rasko DA, et al. Genomic and Phenotypic Diversity among Ten Laboratory Isolates of Pseudomonas aeruginosa PAO1. Journal of bacteriology. 2019;201(5). 10.1128/JB.00595-18 30530517 PMC6379574

[21] Hunstad DA, Justice SS, Hung CS, Lauer SR, Hultgren SJ. Suppression of bladder epithelial cytokine responses by uropathogenic Escherichia coli. Infection and immunity. 2005;73(7):3999–4006. 10.1128/IAI.73.7.3999-4006.2005 15972487 PMC1168571

[22] LaBauve AE, Wargo MJ. Growth and laboratory maintenance of Pseudomonas aeruginosa. Current protocols in microbiology. 2012;Chapter 6:Unit 6E 1. 10.1002/9780471729259.mc06e01s25 22549165 PMC4296558

[23] Knodler LA. Salmonella enterica: living a double life in epithelial cells. Curr Opin Microbiol. 2015;23:23–31. 10.1016/j.mib.2014.10.010 25461569

[24] Kumar Y, Valdivia RH. Leading a sheltered life: intracellular pathogens and maintenance of vacuolar compartments. Cell Host Microbe. 2009;5(6):593–601. 10.1016/j.chom.2009.05.014 19527886 PMC2716004

[25] Martinez JJ, Mulvey MA, Schilling JD, Pinkner JS, Hultgren SJ. Type 1 pilus-mediated bacterial invasion of bladder epithelial cells. The EMBO journal. 2000;19(12):2803–12. 10.1093/emboj/19.12.2803 10856226 PMC203355

[26] Huang H, Shao X, Xie Y, Wang T, Zhang Y, Wang X, et al. An integrated genomic regulatory network of virulence-related transcriptional factors in Pseudomonas aeruginosa. Nat Commun. 2019;10(1):2931. 10.1038/s41467-019-10778-w 31270321 PMC6610081

[27] Lizewski SE, Lundberg DS, Schurr MJ. The transcriptional regulator AlgR is essential for Pseudomonas aeruginosa pathogenesis. Infection and immunity. 2002;70(11):6083–93. 10.1128/iai.70.11.6083-6093.2002 12379685 PMC130412

[28] Kong W, Zhao J, Kang H, Zhu M, Zhou T, Deng X, et al. ChIP-seq reveals the global regulator AlgR mediating cyclic di-GMP synthesis in Pseudomonas aeruginosa. Nucleic acids research. 2015;43(17):8268–82. 10.1093/nar/gkv747 26206672 PMC4787818

[29] Little AS, Okkotsu Y, Reinhart AA, Damron FH, Barbier M, Barrett B, et al. Pseudomonas aeruginosa AlgR Phosphorylation Status Differentially Regulates Pyocyanin and Pyoverdine Production. MBio. 2018;9(1). 10.1128/mBio.02318-17 29382736 PMC5790918

[30] Belete B, Lu H, Wozniak DJ. Pseudomonas aeruginosa AlgR regulates type IV pilus biosynthesis by activating transcription of the fimU-pilVWXY1Y2E operon. Journal of bacteriology. 2008;190(6):2023–30. 10.1128/JB.01623-07 18178737 PMC2258880

[31] Darzins A, Chakrabarty AM. Cloning of genes controlling alginate biosynthesis from a mucoid cystic fibrosis isolate of Pseudomonas aeruginosa. Journal of bacteriology. 1984;159(1):9–18. 10.1128/JB.159.1.9-18.1984 6330052 PMC215585

[32] Lizewski SE, Schurr JR, Jackson DW, Frisk A, Carterson AJ, Schurr MJ. Identification of AlgR-regulated genes in Pseudomonas aeruginosa by use of microarray analysis. Journal of bacteriology. 2004;186(17):5672–84. 10.1128/JB.186.17.5672-5684.2004 15317771 PMC516850

[33] Whitchurch CB, Erova TE, Emery JA, Sargent JL, Harris JM, Semmler AB, et al. Phosphorylation of the Pseudomonas aeruginosa response regulator AlgR is essential for type IV fimbria-mediated twitching motility. Journal of bacteriology. 2002;184(16):4544–54. 10.1128/jb.184.16.4544-4554.2002 12142425 PMC135261

[34] Morici LA, Carterson AJ, Wagner VE, Frisk A, Schurr JR, Honer zu Bentrup K, et al. Pseudomonas aeruginosa AlgR represses the Rhl quorum-sensing system in a biofilm-specific manner. Journal of bacteriology. 2007;189(21):7752–64. 10.1128/JB.01797-06 17766417 PMC2168728

[35] Wu W, Badrane H, Arora S, Baker HV, Jin S. MucA-mediated coordination of type III secretion and alginate synthesis in Pseudomonas aeruginosa. Journal of bacteriology. 2004;186(22):7575–85. 10.1128/JB.186.22.7575-7585.2004 15516570 PMC524895

[36] Bucior I, Pielage JF, Engel JN. Pseudomonas aeruginosa pili and flagella mediate distinct binding and signaling events at the apical and basolateral surface of airway epithelium. PLoS pathogens. 2012;8(4):e1002616. 10.1371/journal.ppat.1002616 22496644 PMC3320588

[37] Whitchurch CB, Alm RA, Mattick JS. The alginate regulator AlgR and an associated sensor FimS are required for twitching motility in Pseudomonas aeruginosa. Proceedings of the National Academy of Sciences of the United States of America. 1996;93(18):9839–43. 10.1073/pnas.93.18.9839 8790418 PMC38516

[38] Ma S, Selvaraj U, Ohman DE, Quarless R, Hassett DJ, Wozniak DJ. Phosphorylation-independent activity of the response regulators AlgB and AlgR in promoting alginate biosynthesis in mucoid Pseudomonas aeruginosa. Journal of bacteriology. 1998;180(4):956–68. 10.1128/JB.180.4.956-968.1998 9473053 PMC106978

[39] Okkotsu Y, Tieku P, Fitzsimmons LF, Churchill ME, Schurr MJ. Pseudomonas aeruginosa AlgR phosphorylation modulates rhamnolipid production and motility. Journal of bacteriology. 2013;195(24):5499–515. 10.1128/JB.00726-13 24097945 PMC3889618

[40] Frisk A, Schurr JR, Wang G, Bertucci DC, Marrero L, Hwang SH, et al. Transcriptome analysis of Pseudomonas aeruginosa after interaction with human airway epithelial cells. Infection and immunity. 2004;72(9):5433–8. 10.1128/IAI.72.9.5433-5438.2004 15322041 PMC517424

[41] Chugani S, Greenberg EP. The influence of human respiratory epithelia on Pseudomonas aeruginosa gene expression. Microb Pathog. 2007;42(1):29–35. 10.1016/j.micpath.2006.10.004 17166692 PMC1934408

[42] Turner KH, Everett J, Trivedi U, Rumbaugh KP, Whiteley M. Requirements for Pseudomonas aeruginosa acute burn and chronic surgical wound infection. PLoS Genet. 2014;10(7):e1004518. 10.1371/journal.pgen.1004518 25057820 PMC4109851

[43] Damron FH, Oglesby-Sherrouse AG, Wilks A, Barbier M. Dual-seq transcriptomics reveals the battle for iron during Pseudomonas aeruginosa acute murine pneumonia. Sci Rep. 2016;6:39172. 10.1038/srep39172 27982111 PMC5159919

[44] Betin V, Penaranda C, Bandyopadhyay N, Yang R, Abitua A, Bhattacharyya RP, et al. Hybridization-based capture of pathogen mRNA enables paired host-pathogen transcriptional analysis. Sci Rep. 2019;9(1):19244. 10.1038/s41598-019-55633-6 31848386 PMC6917760

[45] Avital G, Avraham R, Fan A, Hashimshony T, Hung DT, Yanai I. scDual-Seq: mapping the gene regulatory program of Salmonella infection by host and pathogen single-cell RNA-sequencing. Genome biology. 2017;18(1):200. 10.1186/s13059-017-1340-x 29073931 PMC5658913

[46] Gellatly SL, Needham B, Madera L, Trent MS, Hancock RE. The Pseudomonas aeruginosa PhoP-PhoQ two-component regulatory system is induced upon interaction with epithelial cells and controls cytotoxicity and inflammation. Infection and immunity. 2012;80(9):3122–31. 10.1128/IAI.00382-12 22710876 PMC3418734

[47] Cigana C, Curcuru L, Leone MR, Ierano T, Lore NI, Bianconi I, et al. Pseudomonas aeruginosa exploits lipid A and muropeptides modification as a strategy to lower innate immunity during cystic fibrosis lung infection. PloS one. 2009;4(12):e8439. 10.1371/journal.pone.0008439 20037649 PMC2793027

[48] Hansen MC, Nielsen AK, Molin S, Hammer K, Kilstrup M. Changes in rRNA levels during stress invalidates results from mRNA blotting: fluorescence in situ rRNA hybridization permits renormalization for estimation of cellular mRNA levels. Journal of bacteriology. 2001;183(16):4747–51. 10.1128/JB.183.16.4747-4751.2001 11466277 PMC99528

[49] Jenner RG, Young RA. Insights into host responses against pathogens from transcriptional profiling. Nature reviews Microbiology. 2005;3(4):281–94. 10.1038/nrmicro1126 15806094

[50] Liberzon A, Birger C, Thorvaldsdottir H, Ghandi M, Mesirov JP, Tamayo P. The Molecular Signatures Database (MSigDB) hallmark gene set collection. Cell Syst. 2015;1(6):417–25. 10.1016/j.cels.2015.12.004 26771021 PMC4707969

[51] Bamborough P, Christopher JA, Cutler GJ, Dickson MC, Mellor GW, Morey JV, et al. 5-(1H-Benzimidazol-1-yl)-3-alkoxy-2-thiophenecarbonitriles as potent, selective, inhibitors of IKK-epsilon kinase. Bioorg Med Chem Lett. 2006;16(24):6236–40. 10.1016/j.bmcl.2006.09.018 16997559

[52] Kobori M, Yang Z, Gong D, Heissmeyer V, Zhu H, Jung YK, et al. Wedelolactone suppresses LPS-induced caspase-11 expression by directly inhibiting the IKK complex. Cell Death Differ. 2004;11(1):123–30. 10.1038/sj.cdd.4401325 14526390

[53] Manna SK, Aggarwal BB. Wortmannin inhibits activation of nuclear transcription factors NF-kappaB and activated protein-1 induced by lipopolysaccharide and phorbol ester. FEBS Lett. 2000;473(1):113–8. 10.1016/s0014-5793(00)01501-5 10802070

[54] Hung CS, Dodson KW, Hultgren SJ. A murine model of urinary tract infection. Nat Protoc. 2009;4(8):1230–43. 10.1038/nprot.2009.116 19644462 PMC2963178

[55] Justice SS, Lauer SR, Hultgren SJ, Hunstad DA. Maturation of intracellular Escherichia coli communities requires SurA. Infection and immunity. 2006;74(8):4793–800. 10.1128/IAI.00355-06 16861667 PMC1539609

[56] Helaine S, Cheverton AM, Watson KG, Faure LM, Matthews SA, Holden DW. Internalization of Salmonella by macrophages induces formation of nonreplicating persisters. Science. 2014;343(6167):204–8. 10.1126/science.1244705 24408438 PMC6485627

[57] Adams KN, Takaki K, Connolly LE, Wiedenhoft H, Winglee K, Humbert O, et al. Drug tolerance in replicating mycobacteria mediated by a macrophage-induced efflux mechanism. Cell. 2011;145(1):39–53. 10.1016/j.cell.2011.02.022 21376383 PMC3117281

[58] Lamas Ferreiro JL, Alvarez Otero J, Gonzalez Gonzalez L, Novoa Lamazares L, Arca Blanco A, Bermudez Sanjurjo JR, et al. Pseudomonas aeruginosa urinary tract infections in hospitalized patients: Mortality and prognostic factors. PloS one. 2017;12(5):e0178178. 10.1371/journal.pone.0178178 28552972 PMC5446154

[59] Marschall J, Carpenter CR, Fowler S, Trautner BW, Program CDCPE. Antibiotic prophylaxis for urinary tract infections after removal of urinary catheter: meta-analysis. BMJ. 2013;346:f3147. 10.1136/bmj.f3147 23757735 PMC3678514

[60] Rosen DA, Hooton TM, Stamm WE, Humphrey PA, Hultgren SJ. Detection of intracellular bacterial communities in human urinary tract infection. PLoS Med. 2007;4(12):e329. 10.1371/journal.pmed.0040329 18092884 PMC2140087

[61] Hannan TJ, Totsika M, Mansfield KJ, Moore KH, Schembri MA, Hultgren SJ. Host-pathogen checkpoints and population bottlenecks in persistent and intracellular uropathogenic Escherichia coli bladder infection. FEMS microbiology reviews. 2012;36(3):616–48. 10.1111/j.1574-6976.2012.00339.x 22404313 PMC3675774

[62] Mulvey MA, Schilling JD, Martinez JJ, Hultgren SJ. Bad bugs and beleaguered bladders: interplay between uropathogenic Escherichia coli and innate host defenses. Proceedings of the National Academy of Sciences of the United States of America. 2000;97(16):8829–35. 10.1073/pnas.97.16.8829 10922042 PMC34019

[63] Lane K, Andres-Terre M, Kudo T, Monack DM, Covert MW. Escalating Threat Levels of Bacterial Infection Can Be Discriminated by Distinct MAPK and NF-kappaB Signaling Dynamics in Single Host Cells. Cell Syst. 2019;8(3):183–96 e4. 10.1016/j.cels.2019.02.008 30904375

[64] Hannan TJ, Mysorekar IU, Hung CS, Isaacson-Schmid ML, Hultgren SJ. Early severe inflammatory responses to uropathogenic E. coli predispose to chronic and recurrent urinary tract infection. PLoS pathogens. 2010;6(8):e1001042. 10.1371/journal.ppat.1001042 20811584 PMC2930321

[65] Chand NS, Clatworthy AE, Hung DT. The two-component sensor KinB acts as a phosphatase to regulate Pseudomonas aeruginosa Virulence. Journal of bacteriology. 2012;194(23):6537–47. 10.1128/JB.01168-12 23024348 PMC3497526

[66] Davis JH, Rubin AJ, Sauer RT. Design, construction and characterization of a set of insulated bacterial promoters. Nucleic acids research. 2011;39(3):1131–41. 10.1093/nar/gkq810 20843779 PMC3035448

[67] Choi KH, Schweizer HP. mini-Tn7 insertion in bacteria with single attTn7 sites: example Pseudomonas aeruginosa. Nat Protoc. 2006;1(1):153–61. 10.1038/nprot.2006.24 17406227

[68] Hmelo LR, Borlee BR, Almblad H, Love ME, Randall TE, Tseng BS, et al. Precision-engineering the Pseudomonas aeruginosa genome with two-step allelic exchange. Nat Protoc. 2015;10(11):1820–41. 10.1038/nprot.2015.115 26492139 PMC4862005

[69] Shishkin AA, Giannoukos G, Kucukural A, Ciulla D, Busby M, Surka C, et al. Simultaneous generation of many RNA-seq libraries in a single reaction. Nature methods. 2015;12(4):323–5. 10.1038/nmeth.3313 25730492 PMC4712044

[70] Love MI, Huber W, Anders S. Moderated estimation of fold change and dispersion for RNA-seq data with DESeq2. Genome biology. 2014;15(12):550. 10.1186/s13059-014-0550-8 25516281 PMC4302049

[71] Mi H, Muruganujan A, Huang X, Ebert D, Mills C, Guo X, et al. Protocol Update for large-scale genome and gene function analysis with the PANTHER classification system (v.14.0). Nat Protoc. 2019;14(3):703–21. 10.1038/s41596-019-0128-8 30804569 PMC6519457

[72] Subramanian A, Tamayo P, Mootha VK, Mukherjee S, Ebert BL, Gillette MA, et al. Gene set enrichment analysis: a knowledge-based approach for interpreting genome-wide expression profiles. Proceedings of the National Academy of Sciences of the United States of America. 2005;102(43):15545–50. 10.1073/pnas.0506580102 16199517 PMC1239896

