# Supplementary figures and images for: Dual transcriptional analysis reveals adaptation of host and pathogen to intracellular survival of Pseudomonas aeruginosa associated with urinary tract infection
Source: PLoS Pathog. 2021 Apr 26;17(4):e1009534. doi: 10.1371/journal.ppat.1009534 (PMC8102004; doi:10.1371/journal.ppat.1009534)

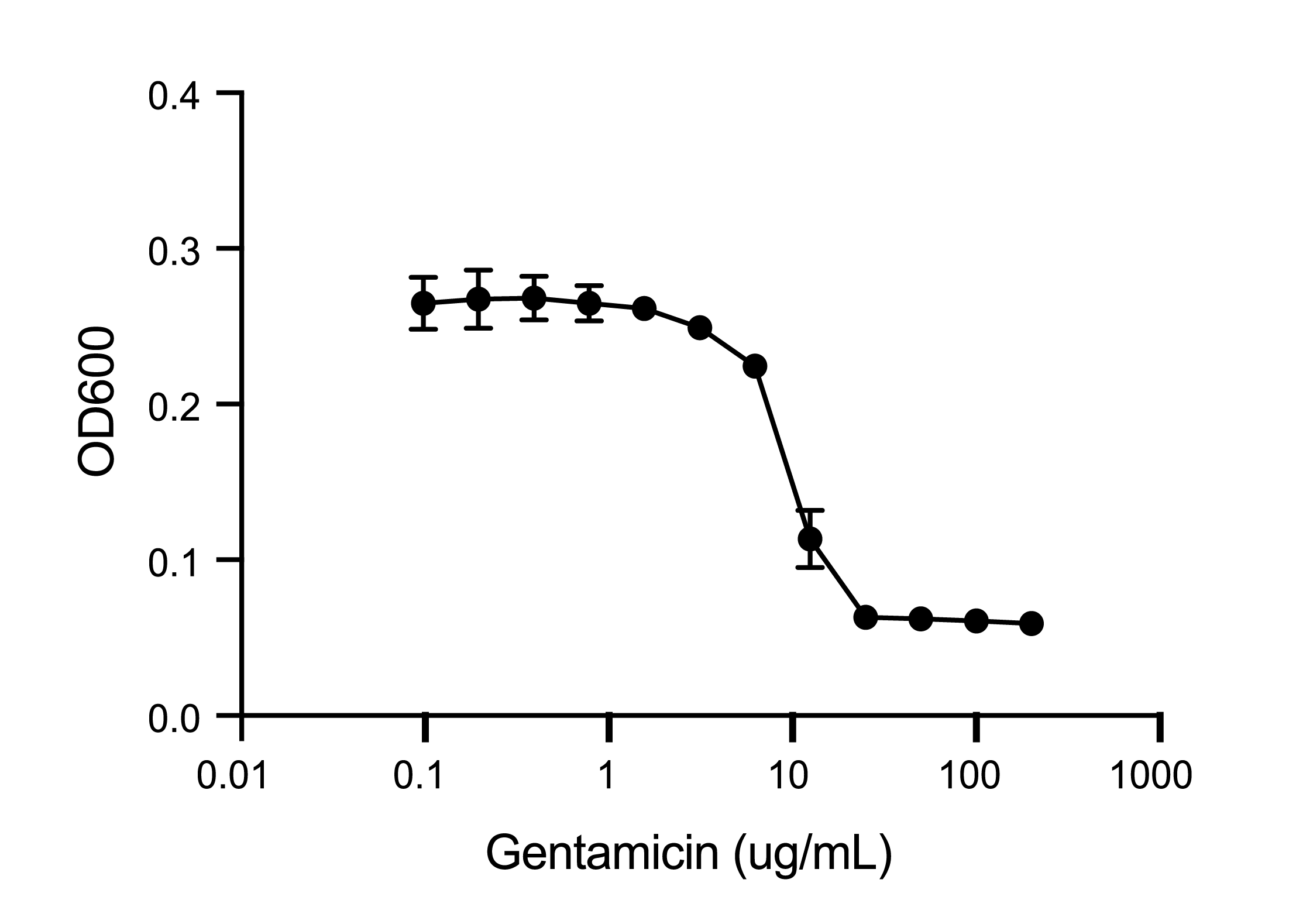

Supplement: S1 Fig — PAO1 was incubated in RPMI + 10% FBS with increasing concentration of gentamicin. OD600 was measured at 16 hours. (TIF) [file ppat.1009534.s001.tif]

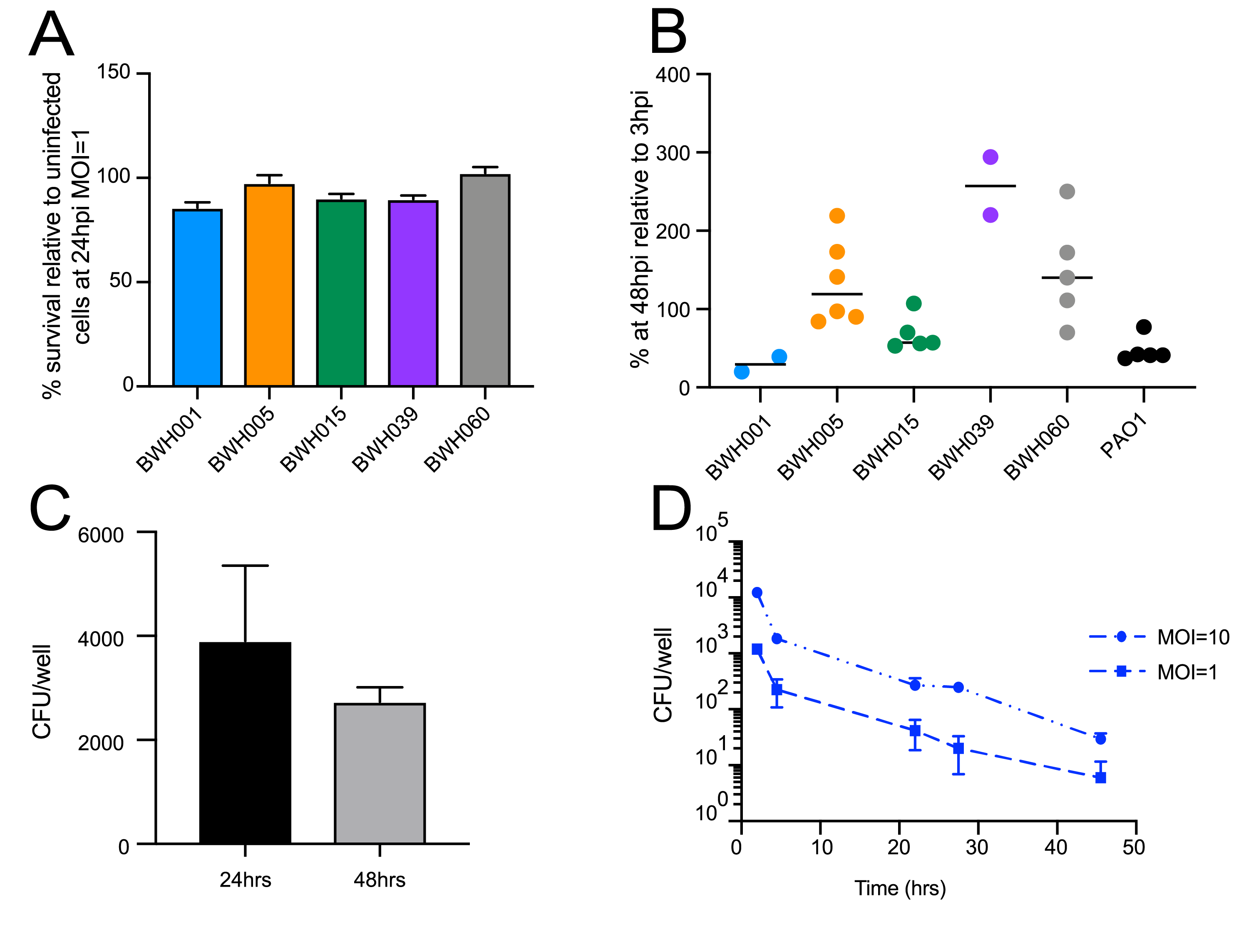

Supplement: S2 Fig — (A-B) Cells were infected with clinical strains isolated from urine at MOI = 1. (A) Host cell survival was measured 24 hpi relative to uninfected cells harvested at the same time point. (B) Percentage of intracellular bacteria recovered at 48 hpi relative to 3 hpi. Each data point represents an independent experiment. (C) A549 lung epithelial cells were infected with WT PAO1. Number of surviving intracellular bacteria were determined at 24 and 48 hpi. Average of two independent experiments is shown. (D) The macrophage-like cell line J774 was infected with WT PAO1. Number of surviving intracellular bacteria were determined at 24 and 48 hpi. Data shown is representative of three independent experiments. (TIF) [file ppat.1009534.s002.tif]

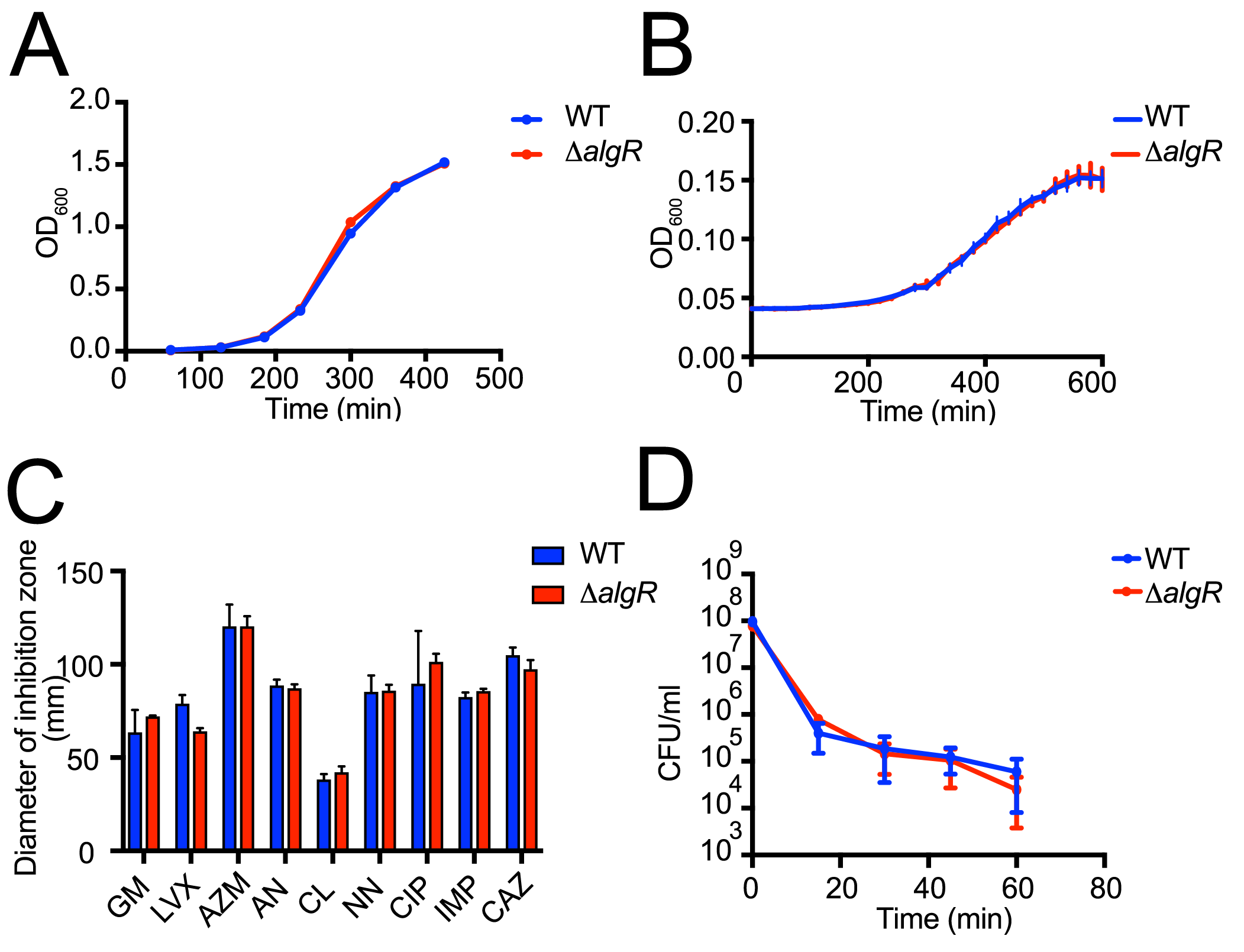

Supplement: S3 Fig — (A, B) Growth of WT and ΔalgR was followed at 37°C in either LB (A) or M9 minimal medium (B) by measuring OD600. (C) Antibiotic susceptibility to gentamicin (GM), levofloxacin (LVX), azithromycin (AZM), amikacin (AN), colistin (CL), tobramycin (NN), ciprofloxacin (CIP), imipenem (IMP) and ceftazidime (CAZ) was determined by disc diffusion. Average zone of inhibition for three discs is shown. (D) Survival of WT and ΔalgR after exposure to 25μM NaOCl in PBS + 10mM glucose determined by plating for CFU. (TIF) [file ppat.1009534.s003.tif]

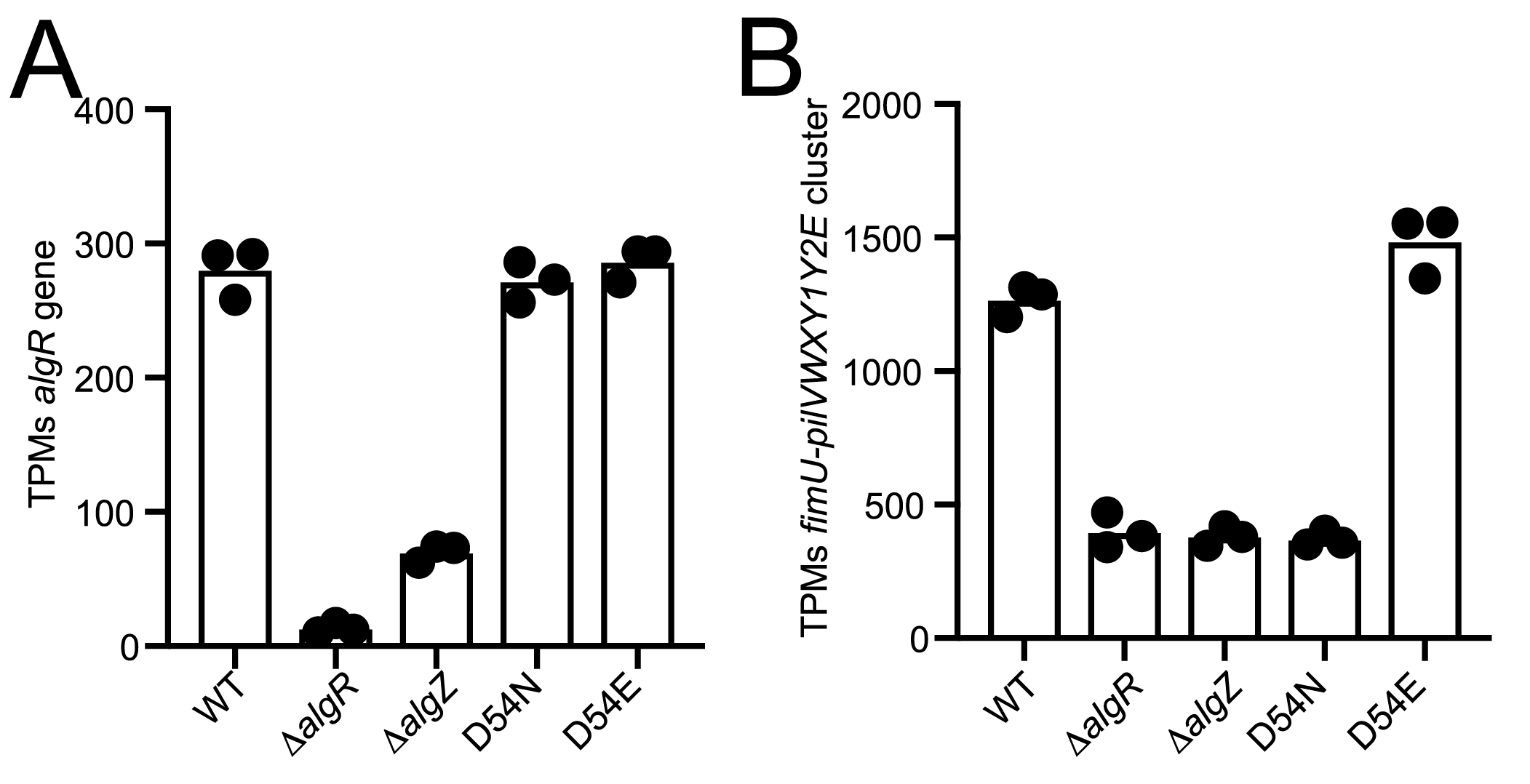

Supplement: S4 Fig — RNA-seq libraries were made of WT, ΔalgR, ΔalgZ and D54N and D54E algR point mutants grown in LB during logarithmic phase. Transcripts per million (TMPs) for (A) algR gene and (B) the sum of the genes in the fimU operon (fimUpilVWXY1Y2E). (TIF) [file ppat.1009534.s004.tif]

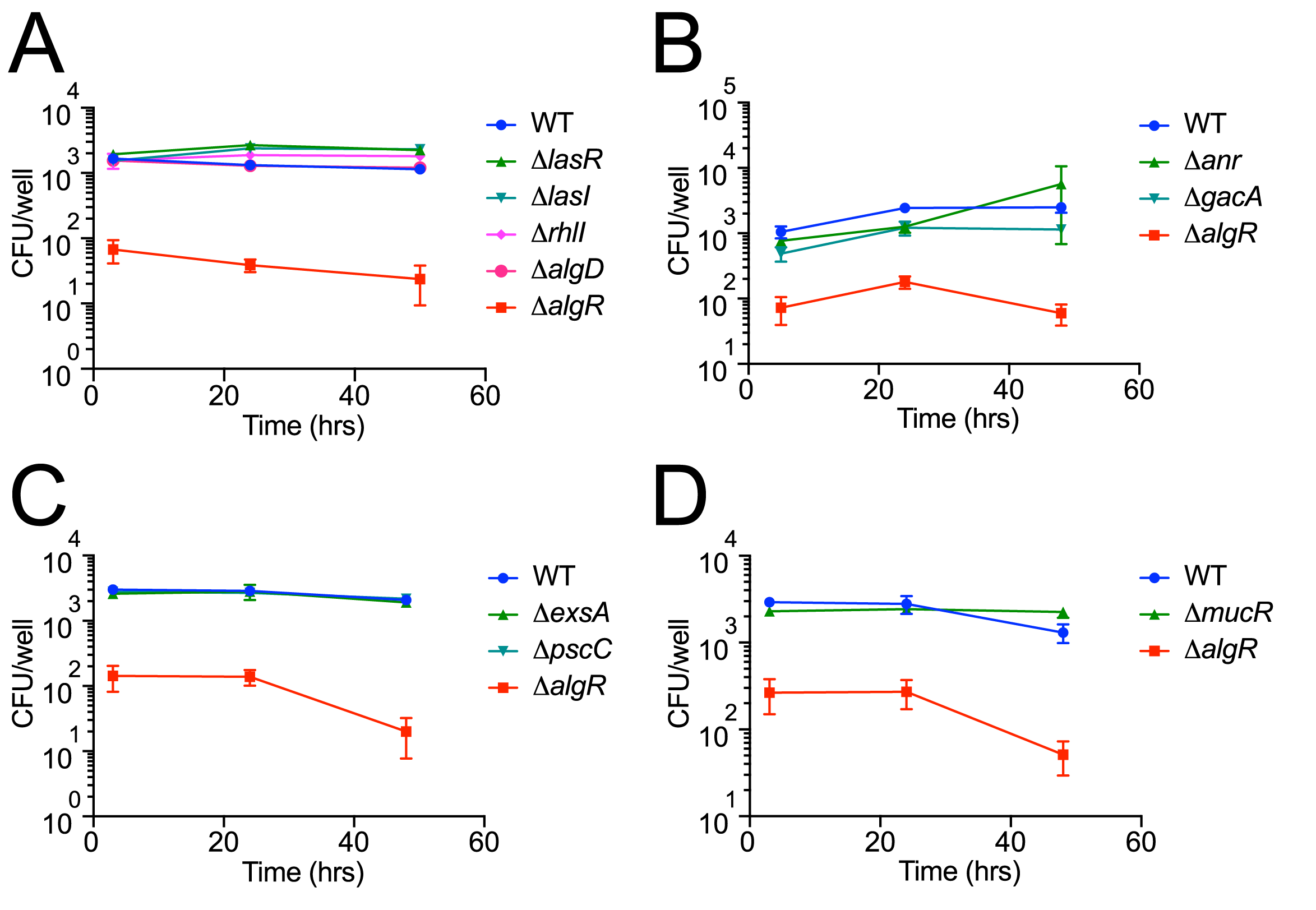

Supplement: S5 Fig — In-frame nonpolar deletions of known AlgR targets were made in the PAO1 background. Cells were infected and harvested at the indicated time points. (A) Quorum sensing regulator lasR and inducers lasI and rhlI. Alginate producing enzyme algD. (B) Transcriptional activator of anaerobic gene expression anr and two-component regulator controlling quorum sensing gacD. (C) Type III secretion regulator exsA and needle component pscC. (D) Alginate biosynthesis regulator mucR. (TIF) [file ppat.1009534.s005.tif]

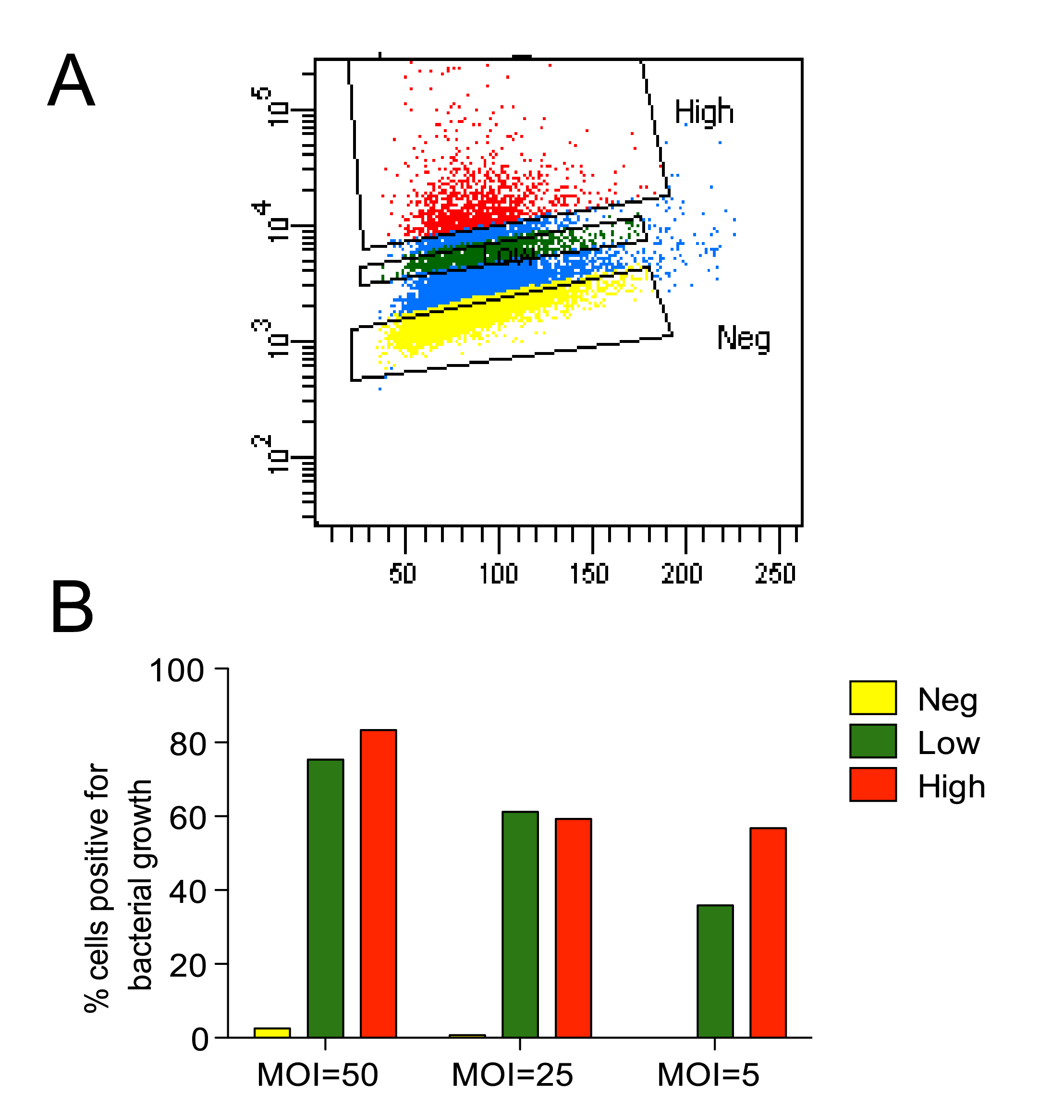

Supplement: S6 Fig — Cells were infected at various MOIs with the GFP expressing WT strain. At 4 hpi cells were harvested and single cells were sorted directly onto agar plates. (A) Gating scheme based on side scatter (SSC) and GFP fluorescence. (B) Plates were incubated overnight and the number of colonies, relative to the number of single cells sorted was determined. For MOI = 50 and 25, 162 cells were sorted per gate. For MOI = 5, 81 cells were sorted per gate. Representative of two independent experiments. (TIF) [file ppat.1009534.s006.tif]

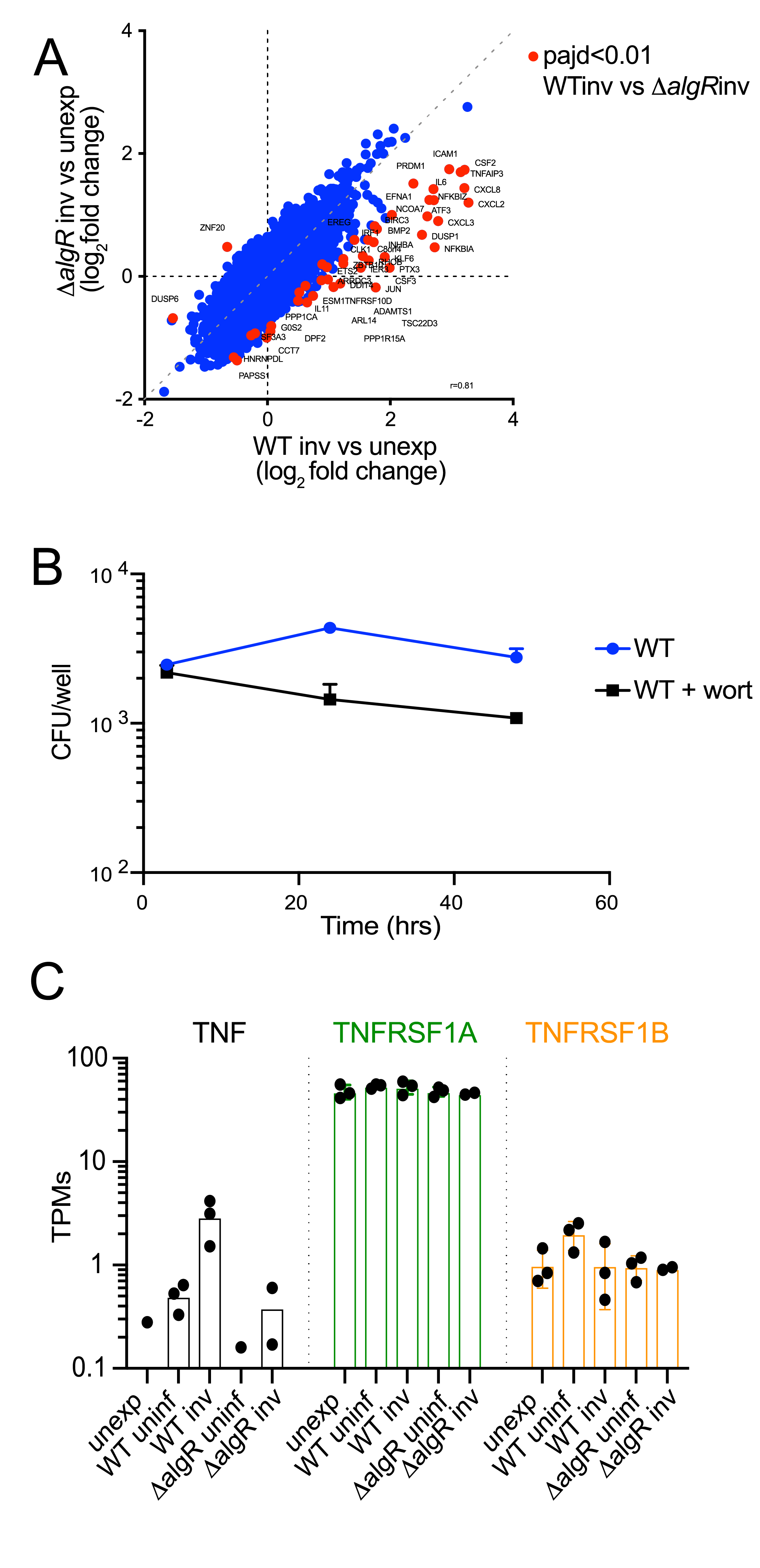

Supplement: S7 Fig — (A) Log2 fold change gene expression of WT-invaded and ΔalgR-invaded cells compared to unexposed cells. Genes differentially expressed between WT-invaded and ΔalgR-invaded cells highlighted in red. (B) Cells were pre-treated for 60 min with wortmannin before infection with WT P. aeruginosa at MOI = 10. At indicated time points the number of intracellular bacteria was determined. (C) Transcripts per million (TMPs) for genes encoding TNFα and its receptors, TNFRSF1A and TNFRSF1B, from the indicated sorted populations. Each data point represents a biological replicate. (TIF) [file ppat.1009534.s007.tif]
