# Supplementary material for: Dual transcriptional analysis reveals adaptation of host and pathogen to intracellular survival of Pseudomonas aeruginosa associated with urinary tract infection
Source: PLoS Pathog. 2021 Apr 26;17(4):e1009534. doi: 10.1371/journal.ppat.1009534 (PMC8102004; doi:10.1371/journal.ppat.1009534)
Supplement: S1 Table — Dual RNAseq libraries were made with the scDualseq protocol and were sequenced before and after enrichment of P. aeruginosa mRNA derived transcripts with PatH-Cap. Percentage of total aligned reads that mapped to the P. aeruginosa genome and specifically to P. aeruginosa mRNA are shown. *of total aligned reads (host + bacteria aligned). (DOCX) [file ppat.1009534.s008.docx]

|  | Pre-PatH-Cap | | | Post-PatH-Cap | | |
| --- | --- | --- | --- | --- | --- | --- |
|  | % PsA* | % PsA mRNA* | Total unique PsA mRNA reads | % PsA* | % PsA mRNA* | Total unique PsA mRNA reads |
| WT_1 | 2.16 | 0.11 | 3,596 | 30.2 | 16.42 | 52,981 |
| WT_2 | 1.80 | 0.11 | 9,027 | 24.0 | 13.72 | 125,164 |
| WT_3 | 2.15 | 0.13 | 9,578 | 29.3 | 17.18 | 137,685 |
| *ΔalgR*_1 | 0.41 | 0.06 | 473 | 11.8 | 6.25 | 4,407 |
| *ΔalgR*_2 | 0.47 | 0.05 | 581 | 8.3 | 5.27 | 4,946 |
| *ΔalgR*_3 | 0.47 | 0.07 | 1,629 | 16.3 | 9.71 | 19,167 |
