# Supplementary material for: Dual transcriptional analysis reveals adaptation of host and pathogen to intracellular survival of Pseudomonas aeruginosa associated with urinary tract infection
Source: PLoS Pathog. 2021 Apr 26;17(4):e1009534. doi: 10.1371/journal.ppat.1009534 (PMC8102004; doi:10.1371/journal.ppat.1009534)
Supplement: S2 Table — Primers used for in-frame nonpolar deletions, algR complementation and site-directed mutagenesis. (DOCX) [file ppat.1009534.s009.docx]

| **Primer** | **Sequence** |
| --- | --- |
| PA4525(pilA)_5p_left | tacaaaaaagcaggctcatttcgaagagggtgtcgt |
| PA4525(pilA)_5p_right | tacaagaaagctgggtctcgtagggctcgaagtgca |
| PA4525(pilA)_3p_left | tcatcttacggaacgttgtgattgggttcctct |
| PA4525(pilA)_3p_right | cacaacgttccgtaagatgaatctctccgttg |
| PA1430(lasR)_5p_left | tacaaaaaagcaggctgatctgggtcttggcattgag |
| PA1430(lasR)_5p_right | ctatgcttaattaatcccgccgcgtagcggccat |
| PA1430(lasR)_3p_left | cgggattaattaagcatagcgctacgttcttctt |
| PA1430(lasR)_3p_right | tacaagaaagctgggtctggatcaacatggtcacctc |
| PA1432(lasI)_5p_left | tacaaaaaagcaggctggtcgcctatctcggtatcag |
| PA1432(lasI)_5p_right | agatgcttaattaatgacggggacctgtcggctc |
| PA1432(lasI)_3p_left | cgtcattaattaagcatcttcacttcctccaaat |
| PA1432(lasI)_3p_right | tacaagaaagctgggtggaaccgtccatctaccagac |
| PA1544(anr)_5p_left | tacaaaaaagcaggctgcatcgacatcacgtcttcc |
| PA1544(anr)_5p_right | cttcattaattaagcattgaggggtccttgctag |
| PA1544(anr)_3p_left | caatgcttaattaatgaagcgcctgcgaaccgcc |
| PA1544(anr)_3p_right | tacaagaaagctgggtctcctcggcatattcgttgtag |
| PA1713(exsA)_5p_left | tacaaaaaagcaggctcagtagaagtgatcctgtgcctg |
| PA1713(exsA)_5p_right | atatgcttaattaatgacgttttttgaaagcccg |
| PA1713(exsA)_3p_left | cgtcattaattaagcatattataagaaccccaac |
| PA1713(exsA)_3p_right | tacaagaaagctgggtggcaaggtccagttgtatgc |
| PA1716(pscC)_5p_left | tacaaaaaagcaggctgtcgccgagcactacatagg |
| PA1716(pscC)_5p_right | tgcctcttaattaaaaggacggcgcctggtgcgt |
| PA1716(pscC)_3p_left | tccttttaattaagaggcagcagcgccagcagtc |
| PA1716(pscC)_3p_right | tacaagaaagctgggtgtctatggctggtgggaaag |
| PA1727(mucR)_5p_left | tacaaaaaagcaggctgccactggaagatcgagaag |
| PA1727(mucR)_5p_right | gctcattaattaagcatgaaagcgtccgaacggg |
| PA1727(mucR)_3p_left | tcatgcttaattaatgagcgccgccagcggcgcg |
| PA1727(mucR)_3p_right | tacaagaaagctgggtctcatgtccacggtgttgtagtc |
| PA2586(gacA)_5p_left | tacaaaaaagcaggctgcttcatccagatcagccc |
| PA2586(gacA)_5p_right | agcagttaattaagcacgctgcacctcgtcgcgc |
| PA2586(gacA)_3p_left | gcgtgcttaattaactgctcgccgtccgccacgg |
| PA2586(gacA)_3p_right | tacaagaaagctgggtaggtgtacgcaagcgcc |
| PA3476(rhlI)_5p_left | tacaaaaaagcaggcttactgcaatgaggaatgacgg |
| PA3476(rhlI)_5p_right | cctcattaattaagcatgaccaagtccccgtgtc |
| PA3476(rhlI)_3p_left | tcatgcttaattaatgaggtcgtcagccgtttcg |
| PA3476(rhlI)_3p_right | tacaagaaagctgggtccctgtactacttgccgtgc |
| PA3540(algD)_5p_left | tacaaaaaagcaggctcactcgctcatcacgtagcc |
| PA3540(algD)_5p_right | cgatgcttaattaatagcgggccgccccctggcc |
| PA3540(algD)_3p_left | cgctattaattaagcatcgcattcacctcgattg |
| PA3540(algD)_3p_right | tacaagaaagctgggtgctggtattcctcagggacg |
| PA5261(algR)_5p_left | tacaaaaaagcaggctccgttgagtcgcttgttcag |
| PA5261(algR)_5p_right | cgcgacttaattaatgacggcggtcggcggttcg |
| PA5261(algR)_3p_left | cgtcattaattaagtcgcgccagaggttcgtcat |
| PA5261(algR)_3p_right | tacaagaaagctgggtagtggatcgtactgctctcgg |
| PA5262(algZ)_5p_left | tacaaaaaagcaggctgcggtttccagttccttgac |
| PA5262(algZ)_5p_right | cgatgcttaattaactacgctatccatgtgcgcg |
| PA5262(algZ)_3p_left | cgtagttaattaagcatcgacagagtttccgcaa |
| PA5262(algZ)_3p_right | tacaagaaagctgggtgtccacagcaccagctcttc |
| PA5261(algR)_complement_fwd | gggtctaga cga cag gcg tta cac aat a |
| PA5261(algR)_complement_rvs | cgcaagctt tga taa cag ttc gaa ccc ga |
| D54N_SDM_fwd | cgtcctgctgaatatccgcatg |
| D54N_SDM_rvs | atatcgggcttgaggctg |
| D54E_SDM_fwd | tcctgctggaaatccgcatgc |
| D54E_SDM_rvs | cgatatcgggcttgaggc |
| algR_replace_wd | tacaaaaaagcaggct﻿gacgacttcttcattcccga |
| algR_replace_rvs | tacaagaaagctgggtgtatggtgcaagggttccag |
